# A widely used corporate digital-transformation index is not fully measurement-invariant: a construct-validity audit of a text-based composite

**DOI:** 10.3389/fpsyg.2026.1923228

**Published:** 2026-10-14

**Authors:** Wenkai Xu, Felipe Hernández-Perlines

**Affiliations:** 1 School of Economics and Management, Wuchang Shouyi College, Wuhan, China; 2 Research Center for Hubei Agricultural Modernization and Rural Development, Wuhan, China; 3 Department of Business Administration, Faculty of Legal and Social Science, University of Castilla-La Mancha, Toledo, Spain

**Keywords:** China, composite indicators, confirmatory factor analysis, construct validity, digital transformation, measurement invariance, partial invariance, text-based measures

## Abstract

**Background:**

Text-derived composite scores are widely used across the social sciences, but their comparability is usually assumed rather than tested.

**Objective:**

We audit whether a widely used corporate digital-transformation index supports the comparisons drawn from it.

**Methods:**

Using 46,799 firm-year observations from 5,267 Chinese A-share firms (2011–2024), we treat the six published sub-dimensions as indicators of a latent construct. With robust maximum likelihood and firm-clustered standard errors we fit the configural–metric–scalar ladder across ownership and industry, add a longitudinal two-wave model for time, revalidate the partial-invariance search in a held-out half of firms, and compare the index with an independent technology-keyword measure.

**Results:**

A one-factor model fits moderately (CFI = 0.959, RMSEA = 0.064) but represents three sub-dimensions weakly (omega = 0.70), and no admissible lower-dimensional alternative improves on it. Across ownership, metric invariance is retained (change in CFI = –0.009) and a partial-invariance model restores comparability (–0.007); ignoring it overstates the state-vs.-private gap by about a fifth. Industry shows the largest failure (metric –0.036), localized to one loading (–0.008); partial scalar invariance then holds against that model (–0.006) but not cumulatively (–0.014), so the comparison is conditional and fragile. Under it the raw composite and the partially invariant reflective latent model yield sector contrasts of opposite sign (+0.06 vs. –0.13 SD), a divergence produced by one sub-dimension and reproduced in a held-out sample and in a MIMIC model. Over time, the longitudinal model retains partial metric invariance (–0.003); scalar invariance requires freeing four of six intercepts, and the implied change in level then ranges from –0.07 to +2.41 SD across the fifteen two-anchor specifications, three of which satisfy the criterion. The two text-based measures correlate 0.78 as latent constructs and 0.53 as composites, a gap consistent with the composites' limited reliability.

**Conclusion:**

Cross-sectional comparability can sometimes be recovered under explicit partial-invariance models; comparability of levels over time is not identified, because the specifications that fit disagree about the change by more than the change itself. Validity depends on the comparison, the grouping dimension, the preprocessing choice, and the aggregation rule; each should be tested rather than assumed.

## 1 Introduction

A single number now anchors a large empirical literature. Studies of corporate digital transformation in China overwhelmingly rely on a text-based index built from the frequency of digital-economy terms in annual reports, and they enter that index into regressions, comparing its level across firms, across ownership types, and across years (Wu et al., 2021; Zhao et al., 2021; Yuan et al., 2021). The index is convenient and reproducible, and its diffusion has been rapid. It is also an instance of a much larger class. Turning dictionary counts and word frequencies into firm- or document-level construct scores has become a standard measurement strategy across the social sciences (Grimmer and Stewart, 2013; Gentzkow et al., 2019), from the tone of financial disclosures (Loughran and McDonald, 2011) to economic policy uncertainty (Baker et al., 2016) and firm-level political risk (Hassan et al., 2019). The questions we raise are therefore not peculiar to one Chinese index; they bear on whether any such text-derived composite supports the cross-group and over-time comparisons researchers routinely build on it.

Consider two firms in the same year, one a manufacturer and one not, that receive the same digital-transformation score from this index. The natural reading is that they occupy the same position on a common scale. Our results say that reading is not available. The six sub-dimensions that make up the score relate to the underlying construct differently in the two sectors: the environment-support component, for instance, carries a standardized loading that differs by 0.16 across the two groups. Equal scores can therefore be produced by different underlying configurations, and the difference between the two firms' scores mixes a difference in digital transformation with a difference in how the index measures it. Whether that mixture matters is not a philosophical question; it is a testable one, and testing it is what this paper does.

The test has a standard form, and it is worth stating in ordinary language before the technical vocabulary arrives. Measurement invariance is assessed as a ladder of three increasingly demanding questions. *Configural* invariance asks whether the index has the same broad structure in each group: do the same components hang together in the same pattern? *Metric* invariance asks whether those components carry the same meaning, or weight, in each group: does a one-unit change in the underlying construct move each component by the same amount? *Scalar* invariance asks whether observed levels can be compared fairly: do the components have the same starting points, so that a difference in scores reflects a difference in the construct rather than a difference in where the measurement begins? Each rung licenses a different kind of comparison, and a failure at one rung invalidates the comparisons that the rungs above it would have supported. When a rung fails for only one or two components, a *partial* invariance model can sometimes restore the comparison by allowing those components to differ while holding the rest equal; we use such models throughout, and we report exactly which components they free.

A critical literature has grown alongside this practice, but it operates at the level of the *score*. “Digital-washing” studies show that firms facing earnings pressure inflate digital language without commensurate substantive change, so the score can be talked up (Shentu et al., 2024; Zhou et al., 2026; Cui et al., 2025). These are important cautions. They take the index as a quantity that can be biased and ask whether its value is honestly reported.

We ask a prior, structural question: **does this index support the comparisons researchers draw from it?** The question is psychometric. Comparing a score across groups or over time is valid only if the measurement model is *invariant*: the construct must relate to its indicators in the same way (metric invariance) and the indicators must have the same origins (scalar invariance) in each group and period (Meredith, 1993; Vandenberg and Lance, 2000; Putnick and Bornstein, 2016). When scalar invariance fails, an observed difference in the index between, say, state and private firms may reflect a difference in *how the construct maps onto words* rather than a difference in digital transformation itself. This property has never been tested for the index, even though the literature that uses it is built on exactly the comparisons that invariance licenses.

Treating the six published sub-dimensions of the EDT index as indicators, and using 46,799 firm-year observations on 5,267 Chinese listed firms, we report five findings.

1. **A weak single factor**. Internal consistency is modest (ω = 0.70), and a single factor fits only moderately while representing three of the six sub-dimensions poorly. The aggregate mainly reflects strategy, technology, and application language.
2. **Cross-industry non-invariance is the most severe, and the sector contrast depends on the aggregation rule**. Across manufacturing and non-manufacturing firms, even *metric* invariance fails: the loadings themselves differ, so the sub-dimensions are weighted differently by sector. The failure is localized: freeing a single loading restores partial metric invariance, and partial scalar invariance then holds against that model, though not cumulatively, so the resulting comparison is conditional and comparatively fragile. Within that qualification the consequence is not cosmetic: the raw unit-weighted composite and the partially invariant reflective latent model yield sector contrasts of *opposite sign*, and the divergence is produced by the single non-invariant sub-dimension.
3. **Cross-ownership scalar non-invariance, but localized and repairable**. Across state vs. non-state firms the factor structure is comparable (metric invariance retained, ΔCFI = –0.009) while indicator origins differ (scalar fails); the failure concentrates in one sub-dimension (environment), and a partial-invariance model restores defensible latent-mean comparisons, which raw scores overstate by about 20%.
4. **Temporal comparability is highly sensitive to preprocessing and is not established**. Within-year standardization stabilizes the two-group ladder, but it removes year-specific location and scale by construction and so cannot speak to the comparability of published levels. In an explicitly longitudinal two-wave model on the firms observed in both periods, partial metric invariance holds, but scalar invariance requires freeing four of the six intercepts, and the two anchors that remain then determine the answer: across the fifteen possible anchor pairs the estimated change in level ranges from −0.07 to +2.41 standard deviations, and the three that satisfy the fit criterion disagree by a factor of eight. Levels of this index are therefore not comparable across years on present evidence, not because no model fits but because the models that fit do not agree.
5. **Two text-based operationalizations agree at the latent level and diverge as scores, largely through unreliability**. The scoring sub-dimensions and an independent technology-keyword set give latent factors that correlate 0.78 while the composite scores built from them correlate 0.53, a gap broadly consistent with ordinary attenuation given the composites' own reliability. Because the joint model fits only moderately and the two families come from the same documents, we do not read this as excluding an aggregation-specific method effect; we read it as showing that unreliability alone can account for the observed shortfall. The practical consequence stands: which measure a study uses moves its results.

The contribution is diagnostic and descriptive; we make no causal claim. Amid renewed scrutiny of the fundamentals of psychological measurement (Uher et al., 2025), it is timely to apply that scrutiny to a composite imported from outside psychology and now used at scale. We bring the standard psychometric apparatus (construct validity, per Cronbach and Meehl, 1955; Borsboom et al., 2004; MacKenzie et al., 2011; the reflective/formative distinction, per Bollen and Lennox, 1991, and Diamantopoulos and Winklhofer, 2001; and measurement-invariance testing, per Vandenberg and Lance, 2000) to a measure that a large applied literature treats as transparent, and we separate the comparisons that a partial-invariance model can recover from those that the data do not support at all.

## 2 The index and measurement-theoretic background

### 2.1 The index and how it is used

The Enterprise Digital Transformation (EDT) index counts occurrences of digital-economy keywords in firms' annual reports and aggregates them, after scaling, into a single firm-year score (Wu et al., 2021). Variants are used across a large and rapidly growing applied literature as a regressor for outcomes from productivity to innovation (Zhao et al., 2021; Yuan et al., 2021; Wu and Li, 2024; Wang and Tang, 2024). The version we audit decomposes the score into six sub-dimensions (strategy-leading, technology-driven, organization-enabling, environment-supporting, digital achievement, and digital application), each itself a scored quantity. Applied work almost always uses the aggregate and discards the sub-dimensions.

### 2.2 The digital-washing critique operates at the score level

A second literature questions whether the score is honest. Firms under earnings pressure use “hyped words” to appear digital (Shentu et al., 2024); digital language can run ahead of substance (Zhou et al., 2026; Cui et al., 2025). This work's object is the score's *bias*. It does not ask whether the index is a comparable measurement across groups and time.

### 2.3 Construct validity, reflective vs. formative measurement, and invariance

The index is built by counting words, and whether such a composite is best understood as reflective (six manifestations of one latent attribute) or formative (six ingredients that jointly constitute an index) is not settled, and our data cannot settle it. The distinction involves the direction of the relationship between constructs and indicators (Jarvis et al., 2003; Coltman et al., 2008), while the rationale for formative measurement has itself been challenged (Edwards, 2011). What our data can address is narrower and, for the literature that uses the index, more consequential. Applied studies treat the aggregate as a single quantity that is comparable across firms, sectors, and years. That practice presupposes a common measurement model, and a common measurement model is exactly what an invariance ladder tests. We therefore fit reflective confirmatory models not as a claim about what the index *is*, but as a test of what the prevailing use of it *assumes*. One distinction must be kept in view throughout, because our conclusions depend on it: the invariance results below are obtained for a **latent reflective model**, whereas applied studies use the **observed composite score**, and conclusions about the former do not transfer automatically to the latter. Where we make a claim about the latent measurement model we say so; where we make a claim about the published aggregate we say that instead, and in Section 4.5 we quantify the gap between them. A reflective specification is defensible here in the weak sense that it is not grossly misspecified: the one-factor model fits moderately and the descriptive tetrads are near zero. Neither of those facts establishes that the published composite should be interpreted reflectively, and we do not claim that they do. Supplementary Section S1 reports all 15 descriptive tetrads.

### 2.4 The gap and our contribution

Three literatures bear on the index but do not meet. Applied work treats it as a comparable scalar and compares its level across firms, ownership types, sectors, and years (Wu et al., 2021; Zhao et al., 2021; Yuan et al., 2021). The digital-washing literature questions the honesty of that level (Shentu et al., 2024; Zhou et al., 2026; Cui et al., 2025). Cross-group comparisons require attention to measurement equivalence (Steenkamp and Baumgartner, 1998). A mature psychometric literature specifies exactly when a composite may be compared across groups and time (Meredith, 1993; Vandenberg and Lance, 2000; Putnick and Bornstein, 2016; Millsap, 2011). No study has brought the third to bear on the first. The omission is consequential: if the index is not invariant across the partitions researchers compare, then a large body of conclusions (state-vs.-private gaps, sectoral contrasts, and time trends) rests on comparisons the measure does not support. We close this gap with the first configural → metric → scalar invariance audit of the index. Our contribution is threefold: (i) we test invariance across the three partitions that dominate applied use (ownership, time, industry); (ii) we distinguish cross-sectional comparisons that can be partly recovered under partial invariance from temporal comparisons for which the admissible specifications do not yield a stable estimate of change; and (iii) by framing the index as one instance of the broad class of text-derived composites, we offer a portable diagnostic for that class rather than a verdict on a single Chinese measure.

## 3 Materials and methods

### 3.1 Data and sample

We use 46,799 firm-year observations on 5,267 Chinese A-share firms, 2011–2024, from CSMAR, for which the EDT index and its six sub-dimensions are available. The unit of analysis is the firm-year. We impose no outcome-based restrictions. Sample characteristics and indicator descriptives are reported in Table 1.

**Table 1:** Descriptive statistics of the six EDT sub-dimension scores (*N* = 46,799 firm-years).

Sub-dimension	Mean	SD	Skewness	Kurtosis	Min	Max
Strategy-leading	46.94	20.61	0.45	2.04	17.3	100.0
Technology-driven	35.16	14.36	2.20	7.32	25.6	100.0
Organization-enabling	26.22	6.87	2.96	14.87	22.2	91.2
Environment-supporting	39.72	20.17	0.87	2.69	10.3	91.0
Digital achievement	27.35	7.19	1.05	6.56	16.2	86.7
Digital application	36.29	16.32	2.05	6.39	24.9	100.0

Marked right-skew (organization, technology, application) and excess kurtosis motivate the robust MLR estimator.

The source panel contains 59,241 firm-years spanning 2001–2024, and listwise deletion on the six sub-dimension scores leaves 46,799 firm-years from 5,267 firms. That accounting overstates the role of missingness, and we now state it precisely. The missingness is block-structured: all six scores are absent in exactly the same 12,442 rows, and no firm-year has partial coverage of the six indicators, so listwise deletion cannot induce selectivity across indicators. It is also, overwhelmingly, a coverage boundary rather than attrition: 12,192 of the 12,442 dropped rows (98.0%) fall in 2001–2010, before the database begins scoring annual reports on these dimensions. Within the 2011–2024 window the index actually covers, 46,799 of 47,049 firm-years are observed, a coverage rate of 99.5%. The 250 remaining gaps are concentrated in 2024 (233 rows, 4.6% of that year) and are recent listings whose reports had not been scored at extraction. Within-window missing rates are 0.7% for non-state and 0.2% for state firms, and 0.4% vs. 0.6% for non-manufacturing and manufacturing firms. Supplementary Table S21 reports missingness by year, ownership, and sector and compares observed with unobserved firm-years on size, leverage, profitability, board composition, institutional ownership, and listing age: the unobserved firm-years are smaller and much more recently listed, as one expects of new listings, but they are half a per cent of the window. An inverse-probability-weighted sensitivity analysis (Supplementary Table S22) reproduces every invariance decision reported below to within 0.001 in ΔCFI and every latent-mean estimate to within 0.006 SD. The panel is unbalanced: 5,267 firms over 14 years give 73,738 possible cells, of which 63.5% are observed. Firms contribute a median of 9 observations (interquartile range 5–14); 1,353 firms are observed in all 14 years and 80 in a single year.

### 3.2 Indicators and standardization

The six sub-dimension scores serve as indicators.^1^ Because they are word-frequency-derived and right-skewed, we standardize each before analysis. For the ownership test we use globally standardized indicators. For the temporal test we report two variants: (i) globally standardized, and (ii) standardized *within year*, which removes year-specific shifts in the mean and dispersion of word counts and therefore isolates change in factor structure. Because that transformation removes location and scale differences by construction, we treat it throughout as a sensitivity analysis of structural stability and not as evidence about the comparability of published levels; the comparison over time is settled instead by the longitudinal design of Section 3.5. A second indicator family (technology word-frequency counts for AI, cloud, big data, and digital-technology application, available for 2021–2024) is reserved for convergent-validity checks.

### 3.3 Grouping variables

Invariance is tested across three partitions central to applied practice: (i) ownership (state vs. non-state), (ii) time period (early ≤ 2017 vs. late ≥2018), and (iii) industry (manufacturing, CSMAR codes C1–C4, vs. other). A separate convergent-validity check (Section 4.6) compares the scoring-based factor with an independent technology-keyword factor available for 2021–2024.

Group sizes are as follows. Ownership: 31,188 firm-years from 3,924 non-state firms and 15,611 firm-years from 1,343 state firms. Industry: 31,088 firm-years from 3,796 manufacturing firms and 15,711 firm-years from 1,766 non-manufacturing firms. Period: 17,077 firm-years from 3,305 firms in 2011–2017 and 29,722 firm-years from 5,244 firms in 2018–2024. Two features of this partitioning bear on interpretation. First, ownership classification is stable within firms: the two ownership groups contain 3,924 and 1,343 firms, summing exactly to the 5,267 firms in the panel, so no firm changes category during the period. Industry classification is not stable in this way, since 295 firms change classification at some point, and Section 4.5 reports that the industry result is unaffected by how those firms are handled. Second, the temporal groups are not independent samples: 3,282 of the 5,267 firms (62.3%) appear in both periods, so the two-group temporal comparison is a comparison of overlapping panels and not a between-subjects design. The non-manufacturing group spans 17 CSRC sections and is correspondingly heterogeneous; its largest single component is information transmission, software and information technology services (3,453 firm-years, 22.0% of the group), so the most digitally intensive sector in the economy is pooled with construction, mining, and real estate. A section-by-section analysis would be preferable, but with the largest non-manufacturing section containing 481 firms and the smallest 12, the resulting group sizes are too unequal to support a multi-group invariance comparison. Supplementary Table S5 gives the full composition.

### 3.4 Analytic strategy

Here, ΔCFI is defined as the CFI of the more constrained model minus that of its reference model; the CFI criterion is therefore ΔCFI ≥−0.01. We assess internal consistency (ω, α, inter-item correlations) and dimensionality (PCA; one- and two-factor CFA), then fit a full multi-group invariance ladder: **configural** (same form, all parameters free across groups), **metric** (equal loadings), and **scalar** (equal loadings and intercepts) (van de Schoot et al., 2012; Fischer and Karl, 2019). Each rung is judged by a decrease in CFI of no more than 0.01 and an increase in RMSEA of no more than 0.015 (Cheung and Rensvold, 2002; Chen, 2007), following current multiple-group guidelines for SEM software (Svetina et al., 2020). Estimation uses **maximum likelihood with robust corrections** (lavaan's estimator = ~MLR~), robust to the indicators' non-normality (cf. Rhemtulla et al., 2012, on robust continuous estimation for non-normal indicators), with **standard errors clustered at the firm** to respect the repeated observation of firms. Where scalar invariance fails, we probe which parameters bind via score tests, to assess whether partial invariance is attainable, and we report standardized loading differences across groups as an effect-size index of non-invariance, complementing the sample-size-sensitive significance tests (in the spirit of dMACS; Nye and Drasgow, 2011). We note two design limits addressed in Section 5.4: the two-group temporal partition contains the same firms in both groups, which is why the temporal comparison is settled by the longitudinal model of Section 3.5 rather than by that partition; and CFA treats firm-years as exchangeable within the clustering adjustment.

Where a rung fails, we do not proceed to the next rung on the constrained model. We first test whether *partial* invariance is attainable at the failed rung, and only then impose the next set of constraints on top of the partial model (Byrne et al., 1989). Loadings are freed one at a time in descending order of the univariate score statistic computed on the constrained model. For the two cross-sectional partitions the intercept released is the pre-specified environment-support candidate, which is also the one the score statistics rank first. Everywhere that hypothesis does not apply (the two-wave longitudinal model of Section 3.5, the one-observation-per-firm checks, and the discovery half of Section 4.7), intercepts are released by the same descending score-statistic rule used for loadings, and the released parameter is named wherever it is reported. **Decision rule**. Each rung is judged by the change in fit relative to the *immediately preceding, less constrained model in the sequence actually estimated*: configural → partial metric → partial scalar when a rung requires partial specification, and configural → metric → scalar otherwise. This is the comparison for which the cut-offs of a decrease in CFI of no more than 0.01 and an increase in RMSEA of no more than 0.015 were calibrated (Cheung and Rensvold, 2002; Chen, 2007), which evaluated successive pairs of nested models rather than cumulative departures from a common baseline. Because a partial model can pass every individual step while still standing some distance from the configural solution, we also report the *cumulative* change relative to each partition's configural model, and we flag any case in which the two references would lead to different conclusions. Both quantities appear for every rung in Table 2. This is a specification search and therefore exploratory. We address it in Section 4.7 with a discovery-validation design in which the specification is selected in one random half of firms, frozen, and then fitted without further search in the untouched half. Estimation uses estimator = ~MLR~ with cluster = ~stkcd~ in lavaan 0.6.21 under R 4.5.2 (semTools 0.5.8). We report the estimator and test terminology exactly as the software produces it rather than under a generic robust-SEM label: for these models lavaan returns estimator = ~ML~, test = ~yuan.bentler.mplus~, se = ~robust.cluster~, and information =~observed~. The reported χ^2^ is therefore the Yuan–Bentler $T_{2}^{*}$ statistic in its Mplus variant, not a Satorra–Bentler scaled statistic, and the standard errors are cluster-robust sandwich estimates; the scaling correction factor for the pooled one-factor model is 3.198. Fit indices are lavaan's robust variants computed from that statistic; SRMR is the standard unscaled value. Reliability ω is compRelSEM; AVE and CR are computed from the standardized solution; HTMT is htmt on standardized indicators. Under se = ~robust.cluster~ lavaan's lavTestScore does not implement a cluster-robust score statistic and falls back to the ordinary one, with a warning; the univariate statistics we use to *order* candidate parameters are therefore not cluster-robust, and we treat them as a ranking device rather than as tests, which is all the freeing rule requires. Complete model syntax and the exact constraint specification for every model reported are given in Supplementary Section S2. All ΔCFI values are reported to four decimal places, since several of the decisions below turn on the third.

**Table 2:** Measurement-invariance fit ladders (robust MLR estimator, firm-clustered SEs; robust fit indices).

Partition/model	χ^2^	df	CFI	TLI	RMSEA	SRMR	ΔCFI (prev.)	ΔCFI (config.)
One-factor (pooled)	542.7	9	0.959	0.932	0.064	0.028	–	–
**Ownership** configural	578.1	18	0.954	0.923	0.068	0.030	–	–
Metric	557.7	23	0.944	0.928	0.066	0.039	–0.0093	–0.0093
**Partial metric** (free Technology loading)	496.2	22	0.953	0.936	0.062	0.032	–0.0009	–0.0009
Scalar	818.7	28	0.914	0.907	0.075	0.048	–0.0308	–0.0401
Partial scalar (intercepts only)	641.1	27	0.937	0.929	0.065	0.041	–0.0079	–0.0172
**Partial scalar** **|partial metric**	574.0	26	0.946	0.937	0.062	0.035	–0.0072	–0.0080
**Industry** configural	768.3	18	0.956	0.927	0.071	0.030	–	–
Metric	1150.5	23	0.920	0.896	0.085	0.057	–0.0361	–0.0361
**Partial metric** (free Environment loading)	748.0	22	0.948	0.929	0.070	0.038	–0.0079	–0.0079
Scalar	5454.1	28	0.624	0.597	0.166	0.123	–0.2960	–0.3321
Partial scalar (intercepts only)	1208.1	27	0.914	0.905	0.081	0.059	–0.0056	–0.0417
**Partial scalar** **|partial metric**	813.4	26	0.943	0.934	0.067	0.041	–0.0056	–0.0135
**Time, global z** configural	1203.1	18	0.924	0.874	0.089	0.041	–	–
Metric	1596.3	23	0.886	0.851	0.097	0.064	–0.0385	–0.0385
**Partial metric** (free Tech, Ach, Env loadings)	1329.9	20	0.912	0.867	0.091	0.049	–0.0128	–0.0128
Scalar	3527.3	28	0.737	0.718	0.133	0.098	–0.1493	–0.1877
**Partial scalar** **|partial metric**	3626.2	25	0.753	0.703	0.137	0.092	–0.1589	–0.1717
**Time, within-year z** configural	322.5	18	0.977	0.961	0.049	0.021	–	–
Metric	459.1	23	0.960	0.948	0.057	0.036	–0.0166	–0.0166
**Partial metric** (free Tech loading)	386.6	22	0.967	0.955	0.053	0.031	–0.0093	–0.0093
Scalar	469.0	28	0.960	0.958	0.052	0.036	+0.0004	–0.0162
**Partial scalar** **|partial metric**	393.5	27	0.968	0.964	0.047	0.031	+0.0004	–0.0089
**Time, two-wave longitudinal** configural	480.8	47	0.975	0.964	0.065	0.041	–	–
Metric	693.4	52	0.961	0.951	0.076	0.064	–0.0132	–0.0132
**Partial metric** (free Technology loading)	529.2	51	0.971	0.963	0.066	0.049	–0.0034	–0.0034
Scalar	2877.2	57	0.828	0.801	0.153	0.207	–0.1333	–0.1465
Scalar | partial metric	2935.7	56	0.828	0.797	0.154	0.207	–0.1432	–0.1467
**Partial scalar** (free Ach, Tech, Env, App intercepts)	539.3	52	0.970	0.962	0.066	0.051	–0.0009	–0.0043

χ^2^ is the Yuan–Bentler $T_{2}^{*}$ statistic that lavaan returns under estimator = ~MLR~; given *N* = 46,799 it is reported for completeness rather than used for testing. Invariance decisions follow a decrease in CFI of no more than 0.01 and an increase in RMSEA of no more than 0.015 (Cheung and Rensvold, 2002; Chen, 2007), applied to the **immediately preceding, less constrained model in the sequence actually estimated**, reported in the column “ΔCFI (prev.)”. For transparency the cumulative change against each partition's own configural model is also given, in the column “ΔCFI (config.)”. Where a rung's predecessor *is* the configural model the two columns coincide. Predecessors are: metric and partial metric → configural; scalar and partial scalar (intercepts only) → metric; partial scalar | partial metric → the corresponding partial-metric model. Rows in bold constitute the adopted sequence for each partition; the fully constrained metric and scalar rows are reported for reference. ΔCFI is given to four decimals because several decisions turn on the third. Every rung that satisfies the ΔCFI criterion also satisfies the ΔRMSEA criterion against the same reference; the largest such ΔRMSEA is +0.003 (time, within-year *z*, partial metric) and most are negative. Freed loadings were selected in descending order of the univariate score statistic on the constrained model; the statistics and a firm-level split-sample cross-validation of that search are in Supplementary Sections S2, S3. The industry partition is the only one in which the two reference columns would lead to different conclusions (see Section 4.5). The two-wave longitudinal block is estimated on the 3,282 firms observed in both periods, one row per firm, with each indicator averaged within firm and period and indicator-specific residuals correlated across occasions (Section 3.5); because there is one observation per firm, no clustering adjustment applies to that block, and its standard errors are the unclustered robust ones. Its partial scalar model satisfies the criterion but rests on only two invariant anchors, and Section 4.4 shows that the latent change it implies is not identified in any useful sense. The two-group temporal blocks are retained above it because they are what a two-group treatment of these data yields, not because they license comparison of levels over time; Section 4.4 explains why they do not.

### 3.5 Longitudinal design for the temporal comparison

The early/late partition is not a between-subjects design: 62.3% of firms appear in both groups, so a two-group comparison adjusts standard errors for the dependence but does not model the repeated-measurement covariance structure that a temporal invariance test is meant to capture. Because the claim at issue is comparability of the same firms over time, we add an explicitly longitudinal analysis and treat it, not the two-group comparison, as the basis for what we conclude about time.

We restrict attention to the 3,282 firms observed in both 2011–2017 and 2018–2024, average each of the six globally standardized indicators within firm and period, and obtain two six-indicator measurement occasions per firm. Averaging within firm and period yields one row per firm, so no clustering adjustment is required. We fit a two-wave confirmatory factor model in which the same latent factor is measured on both occasions and the residual of each indicator is free to correlate with itself across occasions. Identification is by fixed factor: in the configural model the factor variance is set to unity and the factor mean to zero at each occasion, with all loadings and intercepts free; the metric model constrains loadings equal across occasions and frees the second-occasion factor variance; the scalar model additionally constrains intercepts equal and frees the second-occasion factor mean. Where a rung fails we apply the same score-test rule used for the cross-sectional partitions. Full syntax is in Supplementary Section S2.

## 4 Results

### 4.1 Internal consistency and structure

Reliability is modest: ω = 0.70 and α = 0.68 (Table 3); we report McDonald's ω, which (unlike α) does not assume tau-equivalence and is the preferred coefficient here (McDonald, 1999; Revelle and Zinbarg, 2009; Flora, 2020). Convergent validity is weak: the average variance extracted is 0.291, far below the 0.50 convention, and composite reliability is 0.692. The average inter-item correlation is 0.26; strategy-leading correlates 0.44 with technology-driven, but the organization and achievement sub-dimensions are essentially uncorrelated (*r* ≈ 0.00). On the criterion of interchangeable, internally consistent indicators, the index is weak.

**Table 3:** Inter-item correlations, one-factor standardized loadings, and uniqueness.

Sub-dimension	Str	Tec	Org	Env	Ach	App	λ (std.)	Uniqueness
Strategy	1.00						0.643	0.586
Technology	0.44	1.00					0.710	0.496
Organization	0.22	0.29	1.00				0.365	0.867
Environment	0.33	0.27	0.15	1.00			0.409	0.833
Achievement	0.18	0.23	0.00	0.15	1.00		0.297	0.912
Application	0.43	0.48	0.24	0.22	0.21	1.00	0.665	0.558

ω = 0.70, α = 0.68. Mean inter-item *r* = 0.26; organization–achievement *r* ≈ 0.00. All standardized loadings < 1 (no Heywood case).

### 4.2 Dimensionality

PCA returns a first component explaining 39.5% of the variance; four components are needed for 80%, and a second component (eigenvalue 1.00) opposes organization (–0.65) and achievement (+0.75). A one-factor CFA fits moderately under MLR (CFI = 0.959, TLI = 0.932, RMSEA = 0.064, SRMR = 0.028). Standardized loadings range from 0.30 to 0.71 (strategy 0.64, technology 0.71, organization 0.37, environment 0.41, achievement 0.30, application 0.67); all are below 1.0, with no improper (Heywood) solution. Three indicators are poorly represented: the uniqueness of organization (0.87), environment (0.83), and achievement (0.91) implies that the common factor explains only 9%–17% of their variance (Figure 1). The aggregate therefore reflects mainly strategy, technology, and application; organization, environment, and achievement contribute little common variance. A two-factor model splitting enabling indicators (strategy, technology, organization, environment) from realization indicators (achievement, application) does not improve on the one-factor representation: it raises CFI by 0.0003 (0.9596 vs. 0.9593) while *worsening* both TLI (0.9242 vs. 0.9321) and RMSEA (0.0673 vs. 0.0637), and the standardized correlation between the two factors is 0.962 (SE 0.015), so the two are not empirically distinguishable and the additional parameter is not repaid by fit. We therefore retain the one-factor representation, and we emphasize what this does and does not show: it does not show that the index is cleanly unidimensional, since the weak loadings on organization, environment, and achievement rule that out. What it shows is that the enabling/realization partition is not the axis along which these indicators actually separate. The empirically distinct axis, as the principal-components solution indicates, opposes organization to achievement. A descriptive tetrad check is consistent with the reflective specification used in the present audit: across all 15 tetrads of the six standardized indicators the mean absolute tetrad is 0.02 (maximum 0.06), small and consistent with the moderate one-factor fit. We do not read this as evidence favoring a reflective over a formative interpretation; as Section 2.3 states, the data cannot resolve that question, and a formal confirmatory tetrad analysis is left for future work. A reflective CFA is therefore not grossly misspecified, and the invariance results below stand under the reflective reading; a formal confirmatory tetrad analysis (Bollen and Ting, 2000) is the natural next step. Supplementary Tables S6, S7 report both solutions in full.

**Figure 1:**
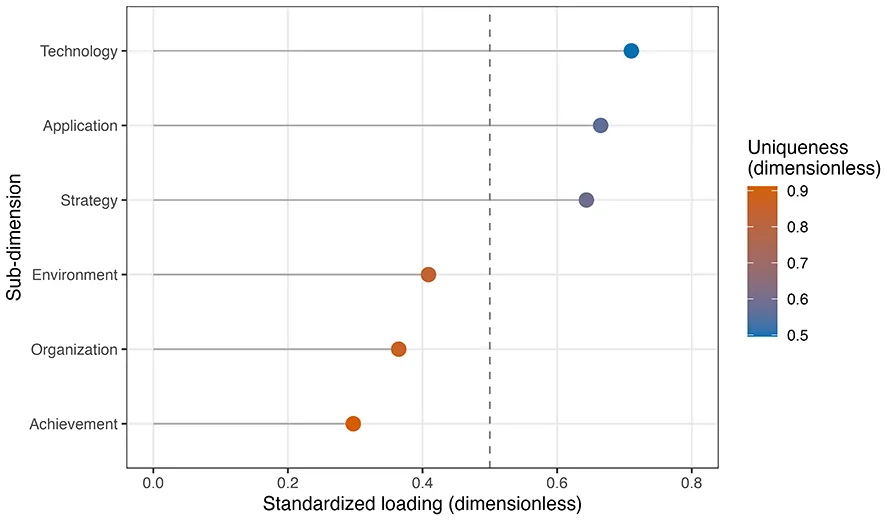
One-factor standardized loadings (lollipop) with indicator uniqueness (color). Organization, environment, and achievement load weakly and have high uniqueness, so the common factor barely represents them.

Two further checks probe dimensionality beyond the principal-components solution. Parallel analysis on the six indicators retains a single component: the first observed eigenvalue (2.372) exceeds its simulated counterpart (1.015), while the second (1.002) does not exceed its own (1.008). It nominally retains four factors on the common-factor criterion, but the additional factors are empirically negligible, with eigenvalues of 0.115, 0.079, and 0.010 against a first factor of 1.749. Splitting the panel at the firm level, an exploratory factor analysis in one half returns at two factors a solution whose second factor is defined by the organization indicator alone (loading 0.997, every other below 0.05); that is a single-indicator factor, not a substantive second dimension. Confirming candidate structures in the held-out half, the one-factor model gives CFI = 0.956, TLI = 0.927, RMSEA = 0.068, SRMR = 0.030; the enabling/realization two-factor model returns a factor correlation of 0.966; and the two-factor structure suggested by the principal-components axis (organization and achievement against the rest) is inadmissible, returning a factor correlation above unity. No admissible low-dimensional alternative improves on the single factor. The conclusion these analyses support is not that the six sub-dimensions are cleanly unidimensional, but that they are only weakly saturated by a common factor, which is the property that governs everything that follows. Supplementary Tables S23, S24 report the parallel analysis and the exploratory solutions.

### 4.3 Measurement invariance across ownership

The full ladder (MLR, firm-clustered; Table 2, Figure 2) begins: configural CFI = 0.9537; metric CFI = 0.9444 (ΔCFI = –0.0093); scalar CFI = 0.9137 (ΔCFI relative to configural = –0.0401). **Metric invariance is retained; scalar invariance fails**. We report the metric change to four decimals deliberately: at two decimals it reads –0.010 and appears to sit exactly on the criterion, but the exact value does not exceed it. The constraint is nonetheless strained, and score tests localize that strain to the technology-driven loading (χ^2^ = 373.3, df = 1), followed by achievement (75.8), organization (34.8), environment (32.7), and application (5.8). Because the metric constraint is strained even though it is not rejected, we also report the partial metric model: freeing the technology loading gives CFI = 0.9529 (ΔCFI = –0.0009 relative to configural), confirming that the residual strain is concentrated in that one parameter. The scalar failure is localized and repairable. Freeing the environment-support intercept alone gives CFI = 0.9365; freeing the technology loading and the environment intercept together, in the model properly nested under the partial metric specification, gives CFI = 0.9457. Judged against the partial metric model under which it is nested, the change is ΔCFI = –0.0072; judged cumulatively against the configural model it is –0.0080. Both references clear the criterion, so nothing in the ownership conclusion depends on which is used. Five of the six sub-dimensions have comparable intercepts across ownership, and latent-mean comparisons of state vs. private firms are defensible under this partial-invariance model. What that repair is worth can be stated in numbers. Estimated on the raw unit-weighted composite, non-state firms exceed state firms by 0.146 standard-deviation units. Under the full scalar model, which our own criterion rejects, the latent difference is 0.150 (SE 0.021). Under the properly nested partial-invariance model it is 0.121 (SE 0.019). Imposing invariance that does not hold thus inflates the estimated ownership gap by 23%, and reading it off raw scores inflates it by 20%. The two partial specifications agree to within one standard error, so the model-based estimate is not sensitive to which is adopted. This is the constructive form of the result: not “no comparison is possible,” but “the comparison requires a partial-invariance model, and skipping it overstates the difference by about a fifth.”

**Figure 2:**
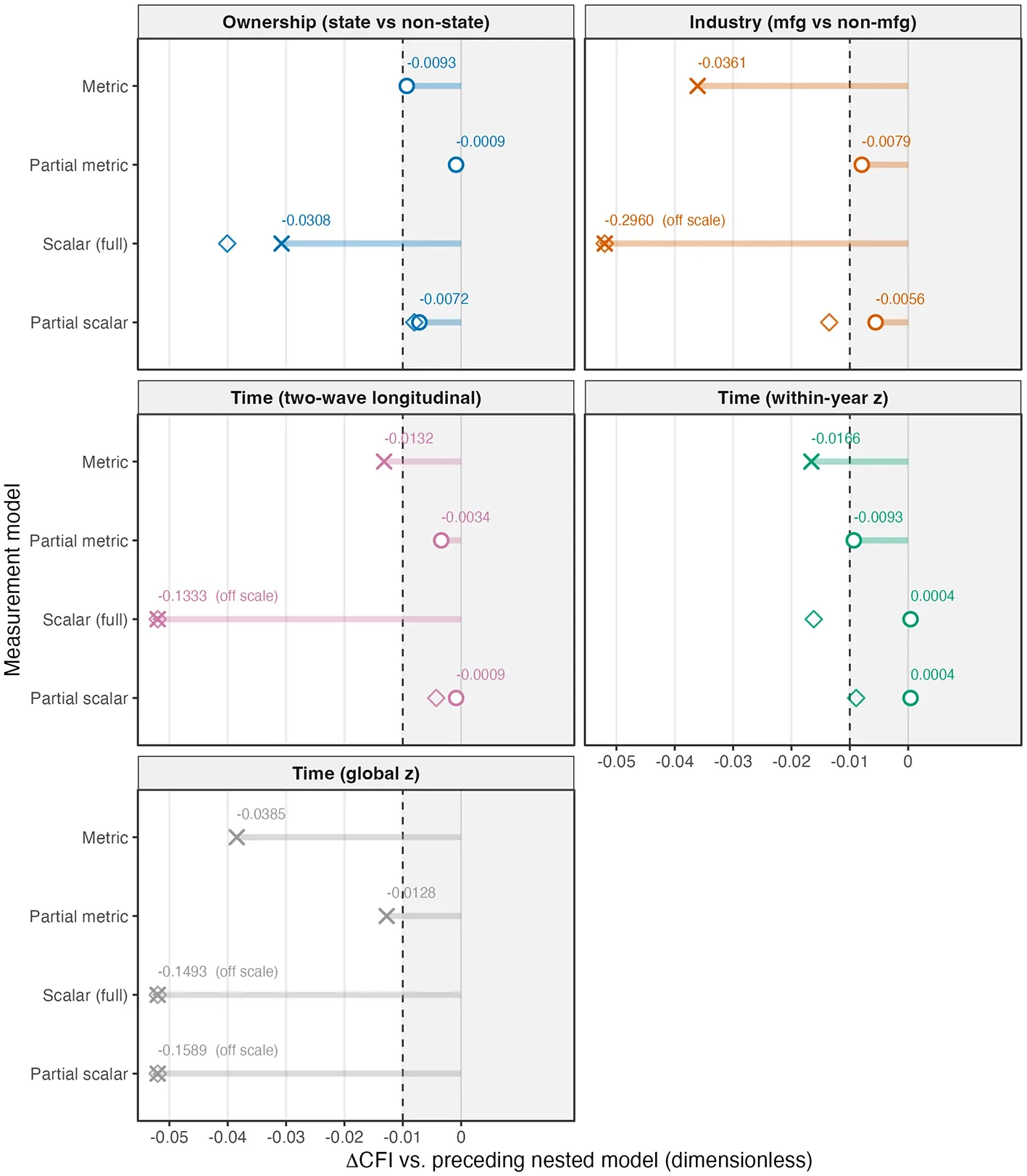
Invariance decisions rest on the change in CFI between nested models. Each bar is the change in robust CFI against the *immediately preceding nested model in the sequence actually estimated* (lavaan estimator = ~MLR~, firm-clustered standard errors), the comparison for which the 0.01 criterion was calibrated; the dashed line is that criterion and the shaded band marks the passing region. Circles pass and crosses fail; bars are truncated at −0.052 and values beyond that are printed and flagged. Open diamonds give the *cumulative* change against the partition's configural model wherever the two references differ; they coincide for rungs whose predecessor is the configural model. Among the cross-sectional partitions, industry is the only one in which the two references lead to different conclusions: its partial-scalar model passes against partial metric (−0.0056) but not cumulatively (−0.0135). The *two-wave longitudinal* panel, estimated on the 3,282 firms observed in both periods with one row per firm (Section 3.5), is the primary evidence on comparability over time: partial metric invariance holds (−0.0034); the fully constrained scalar model fails by an order of magnitude; and the partial scalar model plotted here satisfies the criterion (−0.0009) only after four of the six intercepts are freed, leaving two anchors that, as Section 4.4 shows, determine the latent estimate. The two remaining time panels show what a two-group treatment of the same data yields under global and within-year standardization; Section 4.4 explains why neither licenses comparison of levels. Partial-metric models free one loading, chosen by descending score statistic (ownership, longitudinal, and within-year time: Technology; industry: Environment support); partial-scalar models are nested under the corresponding partial-metric model, whereas the full scalar model is nested under metric and is shown for reference. The number of intercepts freed differs by partition and is given in Table 2. Absolute CFI trajectories appear in Supplementary Figure S1, and the invariance decisions are not based on them.

Three checks bear on the robustness of this conclusion, and they support the direction of the result more firmly than its exact specification. First, because 46,799 firm-years come from 5,267 firms, we repeat the analysis with one observation per firm, averaging each indicator over all available years. This is a between-firm analysis, and ownership is a firm-level attribute, so it is the more direct test of the two. Its configural model fits noticeably worse in absolute terms (CFI = 0.912, RMSEA = 0.119) than the pooled firm-year model. Metric invariance fails (ΔCFI = –0.0205) and is restored by freeing the technology loading (–0.0072), the same indicator the pooled analysis selects. Partial scalar invariance is attainable but requires freeing three intercepts (strategy, organization, and environment) rather than the environment intercept alone (ΔCFI = –0.0025 against partial metric); with fewer freed it does not clear the criterion. The non-state-minus-state contrast under that model is 0.099, against 0.121 in the pooled analysis; the full search is in Supplementary Table S31. Second, inverse-probability weighting for the half per cent of within-window firm-years without index coverage moves the partial-metric ΔCFI from –0.0009 to –0.0012 and the latent contrast from 0.121 to 0.127 (SE 0.019). Third, in the discovery-validation design of Section 4.7 the frozen specification reproduces the sign and rough magnitude in the untouched half (0.107), although the fully constrained metric model fails there (ΔCFI = –0.0113).

What replicates across all three is the direction and approximate size of the ownership gap and the identity of the strained loading. What does not replicate is the exact partial specification: the set of intercepts that must be freed differs between the pooled firm-year analysis and the between-firm analysis. We therefore state the ownership conclusion as follows. Under the pooled firm-year model a limited partial-invariance specification restores comparability, and the raw-score comparison overstates the gap by about a fifth; between firms the same qualitative result holds but requires a larger set of freed intercepts and rests on a configural model with mediocre absolute fit. A multilevel measurement model that separates within-firm from between-firm structure is the appropriate way to settle which specification is right, and we did not fit one; this is recorded in the limitations. The indicator intraclass correlations that motivate the first check are themselves informative and are reported in Supplementary Table S25: between-firm variance accounts for 91.8% of the variance in environment support, 75.1% in strategy, 60.8% in technology, and 50.3% in application, but only 24.9% in organization and 18.0% in achievement. What the index principally records is a stable firm attribute rather than year-to-year movement.

### 4.4 Measurement invariance over time

Two questions must be kept apart here: what happens to the two-group ladder under different preprocessing, and what an explicitly longitudinal model shows. Only the second speaks to whether index levels are comparable over time.

With globally standardized indicators the two-group ladder collapses: configural CFI = 0.9243, metric = 0.8858 (ΔCFI = –0.0385), scalar = 0.7365. Partial metric invariance is not attainable: freeing three loadings in score-test order still leaves ΔCFI = –0.0128 relative to configural. Repeating the ladder with indicators standardized **within year** improves matters substantially: configural CFI = 0.9765, metric = 0.9600 (ΔCFI = –0.0166); freeing the technology loading restores partial metric invariance (CFI = 0.9672, ΔCFI = –0.0093), and scalar constraints imposed on top of that partial model hold (CFI = 0.9676; ΔCFI = +0.0004 against the partial metric model under which it is nested, –0.0089 cumulatively).

That improvement cannot be read as evidence about the comparability of the original scores. After removing year-specific means and dispersions, the remaining covariance structure is substantially more stable across periods. Because year-wise standardization removes location and scale differences by construction, we interpret this result as a sensitivity analysis of structural stability rather than as evidence of scalar invariance of the original score scale. It answers whether the association structure among the sub-dimensions settles once year-specific location and spread are removed; it cannot answer whether levels on the published scale are comparable across years.

The longitudinal model answers the question the two-group ladder cannot. Among the 3,282 firms observed in both periods (Section 3.5), the two-wave configural model fits well (CFI = 0.9746, TLI = 0.9644, RMSEA = 0.0647, SRMR = 0.0408), so the ladder built on it is interpretable. Constraining loadings equal across occasions fails (ΔCFI = –0.0132, ΔRMSEA = +0.0112); freeing the technology loading, the same indicator the two-group analysis selects, restores partial metric invariance (ΔCFI = –0.0034, ΔRMSEA = +0.0015). **Scalar invariance fails severely**: imposing equal intercepts on top of the partial metric model gives ΔCFI = –0.1432. The failure is concentrated but extensive. Score tests reject the equality of four of the six intercepts by wide margins: achievement (χ^2^ = 1524.0), technology (1066.2), environment (800.1), and application (548.2). Strategy (4.9) and organization (4.8) are comparable across occasions.

A partial scalar solution is therefore attainable, and we report it rather than stopping the search early. Freeing the four offending intercepts in score-test order brings the model within the criterion (ΔCFI = –0.0009 against partial metric, –0.0043 cumulatively; CFI = 0.9704, RMSEA = 0.0665, SRMR = 0.0512), leaving strategy and organization as the two invariant anchors. Formally, then, partial scalar invariance over time holds. **Substantively it does not deliver a comparison**, and the reason is worth stating precisely, because it is a general hazard of partial-invariance modeling that our own cross-sectional results do not encounter.

With only two anchors, the estimated change in latent level is a function of which two indicators are chosen to carry the scale. We fitted all fifteen two-anchor specifications. The estimated latent increase from the early to the late period ranges from −0.070 to +2.411 across them. Three of the fifteen satisfy the ΔCFI criterion against the partial metric model, and they do not agree: anchoring on strategy and organization gives +0.904 (SE 0.028, ΔCFI = –0.0009), anchoring on technology and application gives +0.213 (SE 0.015, ΔCFI = –0.0009), and anchoring on organization and achievement gives +1.759 (SE 0.090, ΔCFI = –0.0093). These are not small differences around a common value; they are an eight-fold range in an estimate that the fit criteria cannot adjudicate. For reference, the raw unit-weighted composite rises by 0.278 indicator-standard-deviation units over the same firms and periods, a figure that lies below the whole passing range.

**We therefore do not report an estimate of temporal change, and we do not treat index levels as comparable across years**. The obstacle is not that no model fits, since several do, but that the models that fit disagree about the quantity of interest by more than the quantity itself. Supplementary Table S29 reports the full ladder, the score tests, and all fifteen anchor specifications.

### 4.5 Measurement invariance across industry: the strongest failure

Across manufacturing (CSRC section C, codes C1–C4; 31,088 firm-years from 3,796 firms) vs. all other firms (15,711 firm-years from 1,766 firms), **metric invariance fails**: configural CFI = 0.9561 → metric CFI = 0.9200 (ΔCFI = –0.0361, well beyond the 0.01 cutoff). This is the largest non-invariance we find, and unlike the temporal case it is not a candidate artifact of word-count drift. The failure is, however, localized rather than pervasive. Freeing a single loading, environment support, restores partial metric invariance (CFI = 0.9482, ΔCFI = –0.0079). A common loading structure across the two sectors is therefore available, but only once that one indicator is allowed to differ. The per-indicator standardized loading differences (Table 4, Figure 3) are larger for five of the six sub-dimensions than the corresponding ownership differences and clearly larger overall, reaching 0.174 (organization) and 0.159 (environment); the exception is strategy, where the industry difference (0.017) is smaller than the ownership difference (0.037). Level comparisons across sectors require the same partial specification, and this is the one partition in which the choice of reference model matters. The fully constrained scalar model fits at CFI = 0.6240 and is rejected outright. The partial scalar model nested under the partial metric specification reaches CFI = 0.9426: judged against the partial metric model under which it is nested the change is ΔCFI = –0.0056, within the criterion, and RMSEA improves from 0.0697 to 0.0674; judged cumulatively against the configural model the change is –0.0135, which exceeds it. Under the decision rule stated in Section 3.4 the comparison is therefore available, but it is the weakest of the three, and we mark that explicitly. Partial scalar invariance is supported under the sequential nested-model criterion, although the cumulative fit deterioration relative to the configural model indicates that the resulting latent-mean comparison should be regarded as conditional and comparatively fragile. Three features compound that judgement: the cumulative departure from configural is roughly seventy per cent larger than for ownership (–0.0080), where the two references agree; the partial specification was reached by a specification search rather than derived from theory; and, as the next paragraphs show, the sector contrast changes sign between the two specifications, so the reading of this partition depends on a model choice that the fit criteria do not settle unambiguously.

**Table 4:** Per-group standardized factor loadings (configural models) and their absolute cross-group difference |Δλ| as an effect-size index of metric non-invariance.

Sub-dimension	Ownership: non-state	Ownership: state	|*Δλ*|	Industry: non-mfg	Industry: mfg	|*Δλ*|
Strategy	0.674	0.637	0.037	0.645	0.662	0.017
Technology	0.676	0.724	0.049	0.783	0.658	0.125
Organization	0.439	0.332	0.107	0.476	0.303	0.174
Environment	0.461	0.377	0.084	0.658	0.500	0.159
Achievement	0.332	0.280	0.052	0.351	0.263	0.089
Application	0.680	0.655	0.024	0.682	0.618	0.064

Standardized loadings from the configural (freely estimated) models. |Δλ| is the absolute cross-group difference; industry differences are larger for five of the six sub-dimensions and clearly larger overall, consistent with the stronger metric non-invariance across sectors; the exception is strategy (0.017 across industry vs. 0.037 across ownership). Scalar non-invariance (indicator intercepts, not tabulated) concentrates in the environment sub-dimension, whose intercept difference is the largest in both partitions; freeing it restores partial scalar invariance across ownership (Table 2). Absolute differences were calculated from unrounded estimates and then rounded to three decimal places.

**Figure 3:**
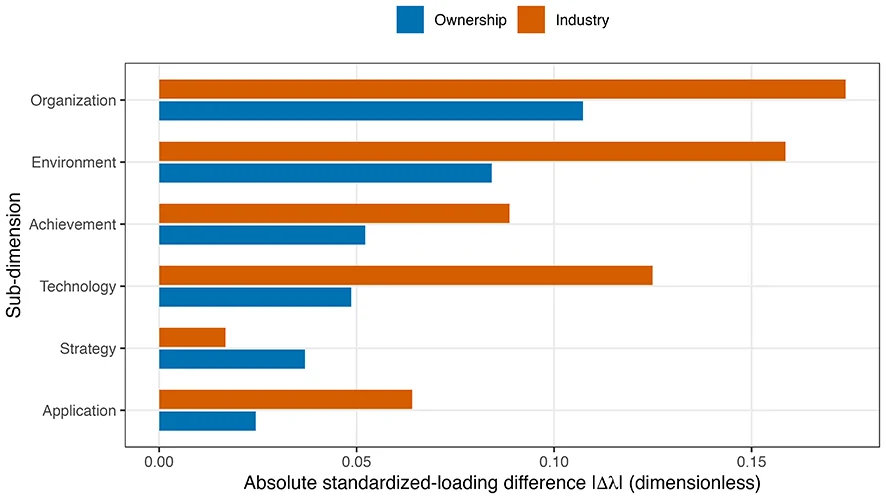
Absolute standardized-loading differences |Δλ| across groups, by sub-dimension, for ownership vs. industry. Industry differences are larger for five of the six sub-dimensions and clearly larger overall, evidencing stronger metric non-invariance across sectors; strategy is the exception.

What the partial specification buys is worth stating precisely, because across sectors the two specifications do not merely differ in the size of the estimated gap; they differ in its sign. **The raw unit-weighted composite and the partially invariant reflective latent-factor model yield sector contrasts of opposite sign**. Under the reflective latent-variable specification adopted for this audit, the estimated manufacturing contrast is –0.131 SD (SE 0.023), whereas the published unit-weighted composite yields +0.063 SD. We state the finding in this form deliberately. As Section 2.3 makes clear, the reflective/formative status of the six sub-dimensions is unresolved, and a reflective latent mean and an observed unit-weighted aggregate need not represent the same estimand; nothing here establishes that the latent estimate is the uniquely correct value of a sector's digital transformation. What the two numbers jointly establish is that the sign of the sector comparison is not a property of the data alone but of the aggregation rule applied to them.

The arithmetic behind the divergence is transparent, and Supplementary Table S26 sets it out indicator by indicator. Manufacturing firms score lower on three of the six sub-dimensions, including the three that load most strongly on the common factor (strategy –0.183, technology –0.164, application –0.122, in standardized units), and are close to indistinguishable on organization (+0.015) and achievement (+0.044). The single exception is environment support, on which they exceed non-manufacturing firms by 0.785. Under equal weights that one indicator contributes +0.131 of the composite's +0.063 total, so every other indicator combined contributes –0.068; under loading weights its contribution falls to +0.104 out of a total of +0.008. The positive sign of the published composite is therefore produced by a single sub-dimension, and it survives only under the equal-weighting rule.

Three specifications that do not share the partial-multi-group form point the same way (Supplementary Table S27). Comparing alternative scores on the same firm-years, expressed in composite standard deviations, the unit-weighted composite gives +0.102, a loading-weighted score gives +0.012, an empirical factor score gives –0.051, and a loading-weighted score that omits environment support gives –0.160. A MIMIC model that regresses the latent factor on sector and allows sector to predict environment support directly, a specification with no group structure at all, estimates the latent sector effect at –0.134 (SE 0.023, *p* < 0.001) with CFI = 0.956, RMSEA = 0.061, SRMR = 0.027, and a direct effect on environment support of +0.877; without that direct path the model fits badly (CFI = 0.769) and the latent effect is indistinguishable from zero (–0.040, *p* = 0.116). The pattern is ordered rather than uniform, and we describe it as such: equal weighting yields a clearly positive contrast; weighting by loadings, which reduces the influence of environment support without removing it, shrinks the contrast to approximately zero (+0.012, *t* = 1.18); and the two specifications that remove or model that indicator's sector dependence, the score omitting it and the empirical factor score, give negative contrasts. The positive manufacturing contrast is therefore specific to equal weighting and is carried by environment support; it does not survive any specification that materially discounts that indicator.

That indicator is also the one that separates the two sectors most sharply. Of the six sub-dimensions, environment support has 13.8% of its variance between the manufacturing and non-manufacturing groups; the next largest share is 0.8% (strategy), and organization and achievement fall below 0.1% (Supplementary Table S19). Whatever this sub-dimension records, it distinguishes sectors far more than it distinguishes firms within them. Because 295 firms change industry classification during the panel, we verified that this conclusion does not depend on how they are treated: under the year-varying classification used above, metric ΔCFI = –0.0361 and the partial metric model reaches –0.0079; excluding the 295 switching firms gives –0.0367 and –0.0091; classifying each firm by its first observed sector gives –0.0244 and –0.0075. The qualitative conclusion is identical under all three treatments. Collapsing to one observation per firm gives the same picture: with each indicator averaged over all available years and sector fixed at its first observed value, metric invariance fails (ΔCFI = –0.0275) and partial metric invariance is restored by freeing the environment and achievement loadings (–0.0082); the partial scalar model passes against it (–0.0091) and estimates the manufacturing contrast at –0.131, while the raw composite on the same one-observation-per-firm data remains positive (+0.042 composite SD). Excluding the 295 switching firms gives –0.116 against a raw +0.042. Inverse-probability weighting gives –0.128. Claims here should be read as applying to the manufacturing vs. non-manufacturing comparison actually tested, and not generalized to sectoral comparisons at large.

### 4.6 Cross-method agreement of two text-based measures

If “digital transformation” were a single construct that firm text simply records, two independent text-based operationalizations of it should agree closely. We compare the six-sub-dimension scoring factor with a four-indicator factor built from independent technology word-frequency counts (AI, cloud, big data, digital-technology application). That family is available from 2021 and the principal panel ends in 2024, so the matched window is 2021–2024: 18,633 firm-years from 4,954 firms drawn from the principal analysis sample. The keyword factor's own measurement properties should be reported before it is used as a comparison standard, and they are not strong: one-factor CFI = 0.978, TLI = 0.934, RMSEA = 0.083 [0.050, 0.121], SRMR = 0.025; standardized loadings 0.763 (big data), 0.677 (AI), 0.525 (cloud), 0.367 (digital-technology application); AVE = 0.363, CR = 0.681, ω = 0.675. AVE below the 0.50 convention mirrors the weakness we document for the scoring-based factor, so neither text-based operationalization is a strong reflective measure and the comparison below should be read accordingly. Three quantities characterize the relationship. The heterotrait–monotrait ratio is HTMT = 0.771 (Table 5), below the conventional 0.85 boundary used to identify empirically distinct factors (Henseler et al., 2015). The correlation between the two latent factors is 0.781 (SE 0.019). The correlation between the unit-weighted composites that researchers actually compute from the two families is 0.535 (Spearman 0.630). Before interpreting these three numbers, the model that produces the first of them should be judged. The joint two-factor model from which the latent correlation is taken fits only moderately [CFI = 0.876, TLI = 0.836, RMSEA = 0.082 (0.077, 0.087), SRMR = 0.049], and its largest modification indices point to structure the two-factor form does not capture: a residual correlation between organization and achievement within the scoring family, a cross-family residual correlation between digital application and the digital-technology-application keyword count, and a cross-loading of the technology sub-dimension on the keyword factor (Supplementary Table S30). The latent correlation of 0.781 is therefore itself a model-dependent quantity, and the comparison that follows should be read as descriptive rather than as a decomposition.

**Table 5:** Reliability and validity of the one-factor model.

Coefficient	Value	Benchmark	Assessment
McDonald's ω	0.70	≥0.70	Borderline
Composite reliability (CR)	0.692	≥0.70	Borderline
Average variance extracted (AVE)	0.291	≥0.50	Fails
HTMT (scoring vs. technology-keyword factor)	0.771	< 0.85 (distinct)	Empirically distinct
Latent correlation (scoring vs. keyword factor)	0.781	–	Constructs closely related
Composite-score correlation (unit-weighted)	0.535	–	Scores only moderately aligned

AVE = 0.291 is well below the 0.50 convention, indicating weak convergent validity within the scoring-based factor; the alternative keyword factor is comparably weak (AVE = 0.363). HTMT = 0.771 is below the conventional 0.85 boundary for empirical distinctness (Henseler et al., 2015). HTMT is a discriminant-validity statistic and not a direct test of agreement, so it is reported here alongside the two quantities that bear on agreement: the latent factor correlation (0.781, SE 0.019) and the correlation between the unit-weighted composites computed from the two families (0.535; Spearman 0.630). Correcting the composite correlation for the two composites' reliability in the matched sample gives 0.810, at or slightly above the latent correlation, so the score-level gap is largely attenuation rather than an aggregation-specific method effect. The two families are close at the construct level; the composites built from them are weak.

The gap between 0.781 and 0.535 is large enough to invite an interpretation it does not support, so we test the obvious alternative first. Two composites of limited reliability cannot correlate as highly as the constructs behind them, and the expected attenuation is computable. Estimated on the matched sample itself, the scoring composite has ω = 0.631 (CR = 0.622, α = 0.587) and the keyword composite ω = 0.691 (CR = 0.685, α = 0.671). Correcting the observed correlation for attenuation gives $r_{\text{corrected}}=0.535/\sqrt{0.631\times 0.691}=0.810$ using ω, or 0.819 using CR, at or slightly above the latent correlation of 0.781. Running the comparison in the other direction, the observed correlation implied by the latent estimate is $0.781\times \sqrt{0.631\times 0.691}=0.515$ (ω) or 0.510 (CR), against an actual 0.535 whose firm-clustered bootstrap 95% interval is [0.513, 0.558]. The residual discrepancy is +0.020 to +0.025 and of the opposite sign to what an additional aggregation-specific method effect would produce.

The conclusion is accordingly narrower than the one we drew previously, and we state it in the terms the numbers license. **Observed score-level agreement is substantially lower than latent agreement, but attenuation analyses indicate that much of this difference is consistent with the limited reliability of the two composite scores**. The point estimates are broadly consistent with ordinary attenuation and provide no evidence here of an additional residual shortfall. We do not go further than that. The two indicator families are drawn from the same annual reports, so independent measurement errors are not a natural assumption; the joint model's modification indices show exactly the kind of cross-family residual association that would violate it; and the bootstrap interval we report covers the observed correlation only, not the reliabilities or the corrected value. These analyses therefore cannot exclude an aggregation-specific method effect, and we withdraw the claim that they establish its absence, as we withdraw the earlier claim, in the opposite direction, that they establish its presence. What survives, and what matters for applied work, is that the scores researchers actually regress share only about 29% of their variance even though the constructs behind them are close. Which text-based digital-transformation measure a study uses remains a researcher degree of freedom, and the reason is now specific: both composites are weak enough that the choice between them moves results.

### 4.7 Discovery and validation of the partial-invariance specifications

Every partial-invariance model reported above was reached by a specification search on the same data used to evaluate it. A firm-level split in which each half searches independently, as reported in the previous version of this work, shows that the search is stable but leaves both halves partly exploratory. We therefore separate discovery from validation outright. Firms, not firm-years, are randomly split in two (2,633 and 2,634 firms; seed fixed and reported with the code). In the discovery half we run the score-test procedure to identify which loadings and intercepts to release. That specification is then *frozen*. In the validation half we fit the frozen model with no modification indices, no score tests, and no further search, alongside the configural and fully constrained models, and we report ΔCFI, ΔRMSEA, and the latent mean difference. Full results are in Supplementary Table S28.

The discovery half selects, for industry, the environment-support loading first, the same indicator identified in the full sample, together with the technology loading, and the environment intercept; for ownership, the environment intercept alone, the metric constraint holding in that half; for the within-year-standardized temporal comparison, the technology and achievement loadings.

One point of interpretation matters and we state it before the results. What is validated is the specification the discovery half selects, which is not in every case the specification the full sample selects: the industry model freezes two loadings where the main-text model frees one, the ownership model freezes none where the main-text model frees the technology loading, and the within-year temporal model freezes two where the main text frees one. This is what a genuine discovery-validation design produces, since the frozen specification must come from the discovery data alone, and it means the exercise validates the *procedure* and the substantive conclusion rather than certifying the exact main-text parameterization. Where the two differ we say so.

In the untouched validation half the frozen specifications behave as the full-sample analysis reports. For industry, configural CFI = 0.9538; the fully constrained metric model fails (ΔCFI = –0.0333); the prespecified partial metric model passes (–0.0040); and the prespecified partial scalar model passes against it (–0.0072) while exceeding the criterion cumulatively (–0.0112), the same divergence between the two references, at nearly the same magnitude, as in the full sample. For ownership, the prespecified partial scalar model passes (–0.0090 against the metric model under which it is nested; –0.0203 cumulatively). ΔRMSEA moves in the same direction as ΔCFI at every prespecified rung in the validation half and never worsens: –0.0026 for the industry partial metric model and –0.0014 for the industry partial scalar model, –0.0008 for the ownership partial scalar model. Most importantly, the substantive result reproduces without any refitting of the specification: the manufacturing-minus-non-manufacturing latent contrast is –0.124 in discovery and –0.138 in validation, against a positive raw composite in both; the non-state-minus-state contrast is 0.132 in discovery and 0.107 in validation. The claim that non-invariance is localized to identifiable indicators, and that modeling it reverses the sign of the sector comparison relative to the unit-weighted composite, therefore does not depend on the sample in which those indicators were identified.

## 5 Discussion

### 5.1 What the audit shows, and what it does not

Table 6 summarizes which comparisons are supported by the measurement results and the corresponding recommendations for using the index. The cautions sort by severity and by remedy, and the two cross-sectional partitions must be separated from the temporal one. Over time, the picture is the most restrictive. The two-group ladder collapses on globally standardized indicators, and within-year standardization repairs it; but that repair removes the year-specific location and scale differences whose comparability is precisely what is in question, so it establishes structural stability and not comparability of levels. The explicitly longitudinal two-wave model on firms observed in both periods fits well and settles the matter differently from either. Partial metric invariance holds after one loading is freed (ΔCFI = –0.0034). Scalar invariance is attainable only by freeing four of the six intercepts, and the two anchors that remain then determine the estimate: across the fifteen possible anchor pairs the implied change in level spans −0.07 to +2.41 SD, and the three pairs that satisfy the fit criterion imply +0.21, +0.90, and +1.76. We therefore withdraw the claim, made in earlier versions of this work, that the temporal alarm is largely mechanical and that partial temporal comparability is supported; but we also decline the opposite overstatement that no partial specification exists. The accurate statement is that a partial specification exists and does not identify the quantity it is wanted for. Cross-sectional comparability can sometimes be partially recovered; temporal comparability of levels cannot be read off this index.

**Table 6:** What the audit licenses, by comparison.

Comparison	Measurement result	Raw comparison defensible?	Recommended remedy
Over time	In an explicitly longitudinal two-wave model on firms observed in both periods, partial metric invariance holds after freeing the Technology loading (ΔCFI = –0.0034); scalar invariance requires freeing four of six intercepts (-0.0009 with four freed), leaving two anchors. The implied change in level ranges from –0.07 to +2.41 SD across the fifteen anchor pairs, three of which satisfy the criterion. Within-year standardization stabilizes the covariance structure but, removing location and scale by construction, cannot establish scalar invariance of the original scale	No—a partial specification exists but does not identify the change	Do not compare index *levels* across years. Analyses of relative within-year standing may use within-year-standardized scores with a partial-invariance model freeing the Technology loading; analyses intended to capture genuine level change require a measurement model with more invariant anchors than these data provide
Across ownership	Metric invariance retained (ΔCFI = –0.0093); scalar fails; nested partial invariance holds (ΔCFI = –0.0072 against partial metric, –0.0080 cumulatively). A between-firm analysis reproduces the direction but needs three intercepts freed rather than one	No—raw scores overstate the gap by about 20%	Partial-invariance latent-mean model freeing the Technology loading and the Environment intercept; report the freed set, which is not stable across the firm-year and between-firm views
Across industry	Metric fails (ΔCFI = –0.0361); partial metric holds after freeing the Environment loading (–0.0079); partial scalar then holds against that model (–0.0056) but not cumulatively (–0.0135), so the resulting latent-mean comparison is conditional and comparatively fragile	No—the raw composite and the partially invariant reflective model give sector contrasts of opposite sign (+0.063 SD vs. –0.131 SD)	Report both estimands and the aggregation rule that produces each; if a single sector contrast is required, fit the partial-invariance model, report the freed parameters, and treat the comparison as the weakest of the three
Scoring-based vs. keyword-based measures	Latent correlation 0.781; composite correlation 0.535; correcting for the composites' reliability (ω = 0.631 and 0.691) gives 0.810, so the score-level gap is broadly consistent with ordinary attenuation; the joint model fits only moderately (CFI = 0.876), so this does not exclude an aggregation-specific method effect	Not as equivalent measures	Justify the choice of measure and report sensitivity to it; treat both composites as low-reliability indicators of a shared construct

Each row states the comparison, what the invariance analysis shows, whether the comparison can be made on raw index values, and what specification is required instead. Full fit statistics are in Table 2.

The cross-sectional results are where recovery is possible. *Cross-industry* non-invariance is the most severe: metric invariance fails (ΔCFI = –0.0361), meaning the loadings, the mapping from construct to words, differ between manufacturing and non-manufacturing firms. The failure is localized: freeing the Environment-support loading restores partial metric invariance (–0.0079), so a common loading structure is available with that one exception made explicit. Measurement origin is the sharper point. The partial scalar model nested under partial metric passes against its own predecessor (–0.0056) while exceeding the criterion cumulatively (–0.0135), the only partition where the two references diverge, so the latent-mean comparison it supports is conditional and comparatively fragile. Within that qualification the substantive result is striking and it replicates: the raw unit-weighted composite and the partially invariant reflective latent model yield sector contrasts of opposite sign, +0.06 SD against –0.13 SD, and the divergence is produced by the single sub-dimension whose invariance fails. A MIMIC model with no group structure, three alternative scoring rules, a one-observation-per-firm analysis, and a frozen specification fitted in a held-out half of firms all reproduce it. We do not claim that the latent estimate is the uniquely correct sector contrast; the reflective/formative status of these indicators is unresolved, and the two numbers are estimands under different aggregation rules. We claim something weaker and more portable: the sign of the sector comparison is a property of the aggregation rule, not of the data alone. *Cross-ownership* non-invariance is milder and repairable: scalar invariance fails, the failure localizes to the environment sub-dimension, and a partial-invariance model that frees that one intercept reinstates defensible latent-mean comparisons, which raw scores overstate by about a fifth. Finally, the two text-based measures are close as constructs (latent correlation 0.781) and only moderately aligned as scores (0.535). The gap is broadly consistent with attenuation due to limited reliability, although the moderate fit of the joint model means that an aggregation-specific method effect cannot be excluded. These cross-sectional results are the audit's substantive contribution.

### 5.2 Relation to prior critiques

Our finding extends the digital-washing literature (Shentu et al., 2024; Zhou et al., 2026; Cui et al., 2025) from the score to the measurement model, and it sits within the broader “construct-audit” genre that has documented divergence and method-dependence in composite ratings: the ESG “aggregate confusion” of Berg et al. (2022), the rater disagreement analyzed by Christensen et al. (2022) and Gibson Brandon et al. (2021), and the more general failure of firm ratings to converge (Chatterji et al., 2016). What that genre shows for survey- and analyst-based composites, we show for a text-derived one, with the added precision of a formal invariance test. The contribution distinct to this paper is the explicit *invariance* test, and the separation of the cross-sectional comparisons a partial-invariance model can partly recover from the temporal comparison, which an explicitly longitudinal model does not support at all.

### 5.3 What valid use requires

Four practices follow, each tied to a specific result. First, for comparisons over time, distinguish the question being asked. Analyses of *relative within-year standing* may use within-year-standardized indicators with a partial-invariance model that frees the Technology loading, reporting the freed parameter. Analyses intended to capture *genuine change in level* should not be run on this index on present evidence. The longitudinal model does admit a partial scalar specification, but only with four of six intercepts freed, and the two anchors that remain determine the answer to within an eight-fold range that the fit criteria cannot narrow; an estimate obtained that way is a statement about the anchors, not about the firms. Year fixed effects are not evidence of measurement invariance and do not substitute for this distinction. Second, for comparisons across ownership, use a partial-invariance latent-mean model that frees the Technology loading and the Environment intercept rather than comparing raw aggregate means, which overstate the gap by about a fifth. Third, for comparisons across industry, do not report a raw aggregate difference on its own: a common loading structure is available only after the Environment-support loading is freed, and the raw composite and the partially invariant latent model give sector contrasts of opposite sign. Report both estimands and the aggregation rule behind each, or, where a single contrast is required, fit the partial-invariance model, name the freed parameters, and flag the comparison as the weakest of the three. More generally, weighting and aggregation choices should be made explicit when constructing and interpreting composite indicators (OECD/European Union/EC-JRC, 2008). Where a sector comparison can be avoided, use sector-specific models or sector-by-construct interactions. Fourth, report and analyze sub-dimensions (especially organization, environment, and achievement, which the aggregate barely represents), and justify the choice between the scoring-based and keyword-based measures rather than treating results as invariant to it. A complementary recommendation falls on data providers: publish the sub-dimensions by default and a within-year-standardized variant. More broadly, the sequence applied here (test the configural → metric → scalar ladder before any group or longitudinal comparison, use an explicitly longitudinal model when the comparison is over time, and quantify residual non-invariance with effect sizes) is a portable check for the wider family of dictionary- and text-derived composites now common across economics, finance, and the behavioral sciences. Invariance is a precondition for comparison wherever such a score is compared across groups or over time (Widaman and Reise, 1997).

### 5.4 Limitations

Five limitations bound what this audit establishes. First, **invariance is not substantive validity**. Our design asks whether the index behaves comparably across selected partitions. It cannot establish that the index captures organizational digital transformation rather than disclosure intensity, rhetorical emphasis, or digital washing. The two properties are independent in both directions: an invariant instrument can measure the wrong attribute uniformly well, and a non-invariant instrument may carry useful information within carefully defined groups. We have no external operational criterion (digital investment, process adoption, technological capability) against which to assess substantive validity, and nothing here supplies one. Second, **the measurement model is one of two defensible readings, and our results are conditional on it**. We audit the index under a reflective specification, in which the six sub-dimensions are effects of a common construct. If they are instead formative components of a defined composite, invariance in the sense tested here is not the relevant criterion and the latent means we report are not the relevant estimand. Section 4.2 shows the data do not settle the question. Everything we report about latent means, and in particular the sector sign reversal, should be read as a statement about what the reflective specification implies relative to the published unit-weighted rule, not as a correction of one by the other. Third, **the partitions are coarse**. The manufacturing vs. non-manufacturing split places 17 CSRC sections, including the information-technology services sector, into a single comparison group; a finer analysis is desirable but is not supported by the resulting group sizes. Fourth, **the temporal analysis leaves the substantive question open, and two design choices bound it**. The two-wave model compares period averages for firms present in both periods. It shows that a partial scalar specification exists but does not identify the change in level, because only two invariant anchors remain and the estimate depends on which two they are. It cannot say how much genuine change underlies the movement we observe, and with two occasions we cannot distinguish a stable trait from a slowly evolving state. A multi-wave model using all years with adequate coverage, or a latent state-trait formulation estimated on more than two occasions, would characterize the trajectory rather than only decline to estimate it; we did not fit one. Separately, the cross-sectional analyses treat firm-years as exchangeable within a clustering adjustment, which corrects inference but does not separate within-firm from between-firm measurement structure; the between-firm results in Section 4.3 differ from the pooled ones in which intercepts must be freed, and a multilevel measurement model is the appropriate way to resolve that difference. Fifth, **setting and data**. The evidence is from Chinese A-share listed firms and the firm-level source data are proprietary, which limits both external generalization and independent verification. The reflective/formative question is examined descriptively; a formal confirmatory tetrad analysis (Bollen and Ting, 2000) is the confirmatory step.

## 6 Conclusion

A single text-based score anchors a large literature on corporate digital transformation. Treated as a measurement instrument, it is a weak single factor whose comparability depends sharply on the partition, and whose cross-sectional failures are localized to identifiable indicators rather than diffuse. Across ownership, comparison is available under a partial-invariance model, and making it on raw scores overstates the difference by about a fifth. Across industry, a common loading structure is available only after the Environment-support loading is freed, and the comparison is the weakest of the three: the raw unit-weighted composite and the partially invariant reflective latent model return sector contrasts of opposite sign, a divergence produced by the single non-invariant sub-dimension and reproduced in a held-out half of firms, in a MIMIC model without group structure, and under three alternative scoring rules. Over time the finding is negative and, we think, the most consequential of the four: in an explicitly longitudinal model on firms observed in both periods, partial metric invariance holds, and a partial scalar specification is attainable only by freeing four of six intercepts, after which the two surviving anchors determine an estimate that ranges over eight-fold across the specifications the fit criteria accept. Index levels are therefore not comparable across years, not for want of a model that fits but because the models that fit disagree about the answer. Finally, the scoring-based and keyword-based measures are close as constructs (latent correlation 0.781) and only moderately aligned as scores (0.535), a gap broadly consistent with the composites' own limited reliability, though the joint model is itself only moderately well fitting. The practical upshot is precise and partition-specific: use partial-invariance latent means for ownership; for industry, report both estimands and the aggregation rule behind each; do not compare index levels across years; and justify the choice of digital-transformation measure rather than assuming results are invariant to it. Getting the measurement right, which means knowing which comparisons the index supports and which it does not, is a precondition for getting the economics of digital transformation right.

## Data Availability

The firm-level data analyzed in this study were obtained from the China Stock Market and Accounting Research (CSMAR) database under license. These third-party data are subject to access and redistribution restrictions and cannot be publicly redistributed by the authors. Researchers may request access directly from CSMAR (https://www.gtarsc.com/), subject to the provider's licensing terms. Questions about the sample construction and analysis may be directed to Wenkai Xu (xuwenkai1226@gmail.com). The analysis code, aggregate statistical outputs and figure-generation materials are publicly available in Zenodo (version 1.0.0): https://doi.org/10.5281/zenodo.22897032. The archive documents the analysis stages, dependencies, input requirements and validation scope. Re-estimation requires independently authorized access to the underlying CSMAR data and reconstruction of the input panel; the archive does not distribute the licensed firm-level data.

## Footnotes

1 The six sub-dimension scores (strategy-leading, technology-driven, organization-enabling, environment-supporting, digital achievement, and digital application) are obtained from the CSMAR (China Stock Market and Accounting Research) Digital Transformation Research Database, which scores the text of listed firms' annual reports along these six dimensions. This scored variant builds on the annual-report text-frequency tradition for measuring corporate digitalization (Wu et al., 2021) and operationalizes the multidimensional view of digital transformation in the literature (Vial, 2019; Verhoef et al., 2021); the six published sub-scores are the database's own operationalization, which the present audit takes as its object. The aggregate index and the six sub-scores share the same firm-year coverage in our sample.

## Supplementary material

Supplementary Data Sheet 1| Supplementary methods, model specifications, robustness and validation analyses, Supplementary Tables S1–S31, and the inventory of reproduction materials.

Supplementary Figure S1| Absolute robust CFI values across measurement models and comparison groups. Invariance decisions are based on changes in fit between nested models, as shown in Figure 2, rather than on absolute CFI alone.
