# Supplementary material for: A widely used corporate digital-transformation index is not fully measurement-invariant: a construct-validity audit of a text-based composite
Source: Front Psychol. 2026 Oct 14;17:1923228. doi: 10.3389/fpsyg.2026.1923228 (PMC13713454; doi:10.3389/fpsyg.2026.1923228)
Supplement: Supplementary file 1 [file Data_Sheet_1.pdf]

# Contents

**Supplementary Material** **2**
§S1 Reflective versus formative measurement: extended treatment . . . 2
Descriptive tetrads . . . 2
Table S18. Descriptive tetrads . . . 2
§S2 Reproduction, model specification, and computational detail . . . 2
S2.1 Reproduction of the originally submitted values . . . 2
Table S1. Reproduction check . . . 2
S2.2 Sample construction, missing data, and panel structure . . . 3
Table S2. Missing-data accounting . . . 3
Table S3. Panel structure . . . 3
Table S4. Stability of group membership within firms . . . 3
Table S5. Industry composition of the two comparison groups . . . 3
S2.3 Dimensionality . . . 4
Table S6. One-factor versus two-factor confirmatory models . . . 4
Table S7. Two-factor model, standardized parameters . . . 4
S2.4 Full invariance ladders including partial-invariance models . . . 5
Table S8. Nested invariance sequence, ownership and industry . . . 5
Table S9. Temporal ladders under both standardizations . . . 6
Table S10. Univariate score tests on metric constraints . . . 6
Table S11. Ownership latent-mean difference under four specifications . . . 6
S2.5 Measurement properties of the alternative keyword factor . . . 7
Table S12. Keyword factor, standardized loadings . . . 7
Table S13. Keyword factor, fit and reliability . . . 7
Table S14. Cross-method agreement . . . 7
§S2.6 Software and reproduction . . . 7
§S3 Cross-validation of the partial-invariance specification search . . . 8
Table S15. Split-sample cross-validation . . . 8
Table S16. Agreement of the independent searches . . . 8
§S4 Robustness to industry-classification changes . . . 8
Table S17. Industry ladder under three classification treatments . . . 8
§S5 Sector comparison under partial invariance (added in the second revision) . . . 9
Table S19. Between-sector variance share of each sub-dimension . . . 9
Table S20. Sector difference in latent means under four specifications . . . 9
§S6 Analyses added in the third revision (Guest Associate Editor) . . . 9
S6.1 Missing data within the index coverage window . . . 9
Table S21. Missingness by year, ownership, and sector, and observed-versus-unobserved balance . . . 9
Table S22. Inverse-probability-weighted sensitivity analysis . . . 10
S6.2 Dimensionality beyond principal components . . . 11
Table S23. Parallel analysis on the six sub-dimensions . . . 11
Table S24. Exploratory solution in the discovery half and confirmation in the validation half . . . 11
S6.3 Panel structure . . . 11
Table S25. Indicator intraclass correlations . . . 11
S6.4 Where the sector contrast comes from . . . 12
Table S26. Indicator contributions to the sector contrast . . . 12
Table S27. Alternative scores and MIMIC specifications . . . 12
S6.5 Discovery and validation . . . 13
Table S28. Frozen specifications fitted in the untouched validation half . . . 13
Table S29. Two-wave longitudinal model, detail . . . 15
Table S30. Joint two-factor model underlying the cross-method comparison . . . 17
Table S31. Ownership, one observation per firm: full intercept search . . . 17
S6.6 Estimator and test terminology as produced by the software . . . 17
S6.7 Contents of the reproduction deposit . . . 18

# Supplementary Material

*A Widely Used Corporate Digital-Transformation Index Is Not Fully Measurement-Invariant: A Construct-Validity Audit of a Text-Based Composite*

Frontiers in Psychology, manuscript 1923228. Third revision, 2026-09-05.

All quantities below are generated directly from the frozen analysis panel by the scripts listed in §S2.6 and §S6.7; no value in this document is transcribed by hand.

---

## §S1 Reflective versus formative measurement: extended treatment

### Descriptive tetrads

All 15 tetrads of the six standardized indicators. These are descriptive; they are not a formal Bollen–Ting confirmatory tetrad test and do not adjudicate the measurement model.

**Table S18. Descriptive tetrads**

| Indicators | Tetrad | Absolute |
|---|---|---|
| strat, tech, org, env | 0.0070 | 0.0070 |
| strat, tech, org, ach | -0.0502 | 0.0502 |
| strat, tech, org, app | 0.0003 | 0.0003 |
| strat, tech, env, ach | -0.0099 | 0.0099 |
| strat, tech, env, app | -0.0596 | 0.0596 |
| strat, tech, ach, app | 0.0021 | 0.0021 |
| strat, org, env, ach | 0.0327 | 0.0327 |
| strat, org, env, app | -0.0301 | 0.0301 |
| strat, org, ach, app | 0.0009 | 0.0009 |
| strat, env, ach, app | 0.0267 | 0.0267 |
| tech, org, env, ach | 0.0440 | 0.0440 |
| tech, org, env, app | -0.0001 | 0.0001 |
| tech, org, ach, app | 0.0055 | 0.0055 |
| tech, env, ach, app | 0.0052 | 0.0052 |
| org, env, ach, app | 0.0318 | 0.0318 |

Number of tetrads: 15. Mean absolute value: 0.020. Maximum absolute value: 0.060.

## §S2 Reproduction, model specification, and computational detail

### S2.1 Reproduction of the originally submitted values

Before beginning this revision we re-derived every quantity reported in the submitted manuscript from the frozen analysis panel. Agreement is exact to machine precision.

**Table S1. Reproduction check**

| Quantity | Reproduced | Submitted | Absolute difference |
|---|---|---|---|
| nobs | 46,799 | 46,799 | 0.0e+00 |
| n_firms | 5,267 | 5,267 | 0.0e+00 |
| cfi_robust | 0.959267 | 0.959267 | 1.1e-16 |
| tli_robust | 0.932112 | 0.932112 | 2.2e-16 |
| rmsea_robust | 0.063665 | 0.063665 | 2.8e-17 |
| srmr | 0.028366 | 0.028366 | 1.7e-17 |

### S2.2 Sample construction, missing data, and panel structure

**Table S2. Missing-data accounting**

| Stage | N |
|---|---|
| panel rows raw | 59,241 |
| rows with complete six items | 46,799 |
| rows dropped listwise | 12,442 |
| na in strategydrivenscoring | 12,442 |
| na in techenablingscoring | 12,442 |
| na in orgenablingscoring | 12,442 |
| na in envenablingscoring | 12,442 |
| na in digitalachievementscoring | 12,442 |
| na in digitalapplicationscoring | 12,442 |

All six sub-dimension scores are absent in exactly the same 12,442 rows; no firm-year has partial coverage of the six indicators, so listwise deletion cannot induce selectivity across indicators.

**Table S3. Panel structure**

| Quantity | Value |
|---|---|
| n firms | 5,267 |
| n obs | 46,799 |
| years covered | 14 |
| balanced panel cells | 73,738 |
| panel completeness ratio | 0.635 |
| obs per firm min | 1 |
| obs per firm p25 | 5 |
| obs per firm median | 9 |
| obs per firm p75 | 14 |
| obs per firm max | 14 |
| obs per firm mean | 8 |
| firms observed all 14 years | 1,353 |
| firms observed once | 80 |

**Table S4. Stability of group membership within firms**

| Classification | Firms total | Single category | Switching |
|---|---|---|---|
| ownership SOE | 5,267 | 5,267 | 0 |
| industry manufacturing | 5,267 | 4,972 | 295 |
| period early late | 5,267 | 1,985 | 3,282 |

**Table S5. Industry composition of the two comparison groups**

| CSRC section | Sector | Group | Firm-years | Firms | Share of group |
|---|---|---|---|---|---|
| C | Manufacturing | manufacturing | 31,088 | 3,796 | 100.0% |
| I | Information transmission, software and IT services | non-manufacturing | 3,453 | 481 | 22.0% |
| F | Wholesale and retail | non-manufacturing | 2,149 | 247 | 13.7% |
| K | Real estate | non-manufacturing | 1,564 | 167 | 10.0% |
| D | Utilities | non-manufacturing | 1,397 | 149 | 8.9% |
| G | Transport and storage | non-manufacturing | 1,274 | 127 | 8.1% |
| E | Construction | non-manufacturing | 1,161 | 142 | 7.4% |
| B | Mining | non-manufacturing | 963 | 99 | 6.1% |
| M | Scientific research and technical services | non-manufacturing | 716 | 133 | 4.6% |

| CSRC section | Sector | Group | Firm-years | Firms | Share of group |
|---|---|---|---|---|---|
| N | Water, environment and public facilities | non-manufacturing | 716 | 113 | 4.6% |
| R | Culture, sports and entertainment | non-manufacturing | 615 | 72 | 3.9% |
| L | Leasing and business services | non-manufacturing | 578 | 91 | 3.7% |
| A | Agriculture | non-manufacturing | 552 | 60 | 3.5% |
| S | Diversified | non-manufacturing | 249 | 60 | 1.6% |
| Q | Health and social work | non-manufacturing | 119 | 18 | 0.8% |
| H | Accommodation and catering | non-manufacturing | 112 | 13 | 0.7% |
| P | Education | non-manufacturing | 74 | 12 | 0.5% |
| O | Resident and other services | non-manufacturing | 19 | 13 | 0.1% |

### S2.3 Dimensionality

**Table S6. One-factor versus two-factor confirmatory models**

| Model | CFI | TLI | RMSEA | SRMR | Scaled chi-square | df |
|---|---|---|---|---|---|---|
| One factor | 0.9593 | 0.9321 | 0.0637 | 0.0284 | 542.75 | 9 |
| Two factors (enabling / realization) | 0.9596 | 0.9242 | 0.0673 | 0.0282 | 523.29 | 8 |

**Table S7. Two-factor model, standardized parameters**

| Parameter | Std. estimate | SE | z |
|---|---|---|---|
| Enable =~ g_strat | 0.6443 | 0.0094 | 68.69 |
| Enable =~ g_tech | 0.7110 | 0.0095 | 74.99 |
| Enable =~ g_org | 0.3661 | 0.0084 | 43.44 |
| Enable =~ g_env | 0.4093 | 0.0121 | 33.69 |
| Realize =~ g_ach | 0.3021 | 0.0129 | 23.46 |
| Realize =~ g_app | 0.6881 | 0.0130 | 52.78 |
| Enable  Realize | 0.9623 | 0.0149 | 64.45 |

### S2.4 Full invariance ladders including partial-invariance models

**Table S8. Nested invariance sequence, ownership and industry**

| Partition | Rung | Freed | CFI | TLI | RMSEA | SRMR | df | Delta CFI vs configural |
|---|---|---|---|---|---|---|---|---|
| Ownership | Configural | – | 0.9537 | 0.9229 | 0.0683 | 0.0300 | 18 | 0.0000 |
| Ownership | Metric | – | 0.9444 | 0.9275 | 0.0663 | 0.0388 | 23 | -0.0093 |
| Ownership | Partial metric | g_tech loading | 0.9529 | 0.9357 | 0.0624 | 0.0319 | 22 | -0.0009 |
| Ownership | Scalar (full) | – | 0.9137 | 0.9075 | 0.0749 | 0.0483 | 28 | -0.0401 |
| Ownership | Partial scalar (intercept only) | g_env intercept | 0.9365 | 0.9295 | 0.0654 | 0.0413 | 27 | -0.0172 |
| Ownership | Partial scalar (given partial metric) | g_tech loading + g_env intercept | 0.9457 | 0.9374 | 0.0616 | 0.0348 | 26 | -0.0080 |
| Industry | Configural | – | 0.9561 | 0.9269 | 0.0709 | 0.0302 | 18 | 0.0000 |
| Industry | Metric | – | 0.9200 | 0.8957 | 0.0846 | 0.0574 | 23 | -0.0361 |
| Industry | Partial metric | g_env loading | 0.9482 | 0.9293 | 0.0697 | 0.0384 | 22 | -0.0079 |
| Industry | Scalar (full) | – | 0.6240 | 0.5972 | 0.1663 | 0.1234 | 28 | -0.3321 |
| Industry | Partial scalar (intercept only) | g_env intercept | 0.9144 | 0.9049 | 0.0808 | 0.0591 | 27 | -0.0417 |
| Industry | Partial scalar (given partial metric) | g_env loading + g_env intercept | 0.9426 | 0.9338 | 0.0674 | 0.0407 | 26 | -0.0135 |

**Table S9. Temporal ladders under both standardizations**

| Partition | Rung | CFI | RMSEA | SRMR | Delta CFI vs configural |
|---|---|---|---|---|---|
| Within-year z | Configural | 0.9765 | 0.0495 | 0.0208 | 0.0000 |
| Within-year z | Metric | 0.9600 | 0.0572 | 0.0360 | -0.0166 |
| Within-year z | Partial metric, freed: tech | 0.9672 | 0.0529 | 0.0308 | -0.0093 |
| Within-year z | Partial scalar (given partial metric) | 0.9676 | 0.0474 | 0.0308 | -0.0089 |
| Global z | Configural | 0.9243 | 0.0890 | 0.0409 | 0.0000 |
| Global z | Metric | 0.8858 | 0.0967 | 0.0637 | -0.0385 |
| Global z | Partial metric, freed: tech | 0.9049 | 0.0902 | 0.0546 | -0.0194 |
| Global z | Partial metric, freed: tech+ach | 0.9099 | 0.0899 | 0.0499 | -0.0144 |
| Global z | Partial metric, freed: tech+ach+env | 0.9115 | 0.0913 | 0.0489 | -0.0128 |
| Global z | Partial scalar (given partial metric) | 0.7526 | 0.1366 | 0.0925 | -0.1717 |

**Table S10. Univariate score tests on metric constraints**

| Partition | Indicator | Score chi-square | df | p |
|---|---|---|---|---|
| ownership | Technology | 373.28 | 1 | <0.001 |
| ownership | Achievement | 75.77 | 1 | <0.001 |
| ownership | Organization | 34.82 | 1 | <0.001 |
| ownership | Environment | 32.72 | 1 | <0.001 |
| ownership | Application | 5.83 | 1 | 0.016 |
| industry | Environment | 1297.05 | 1 | <0.001 |
| industry | Technology | 267.34 | 1 | <0.001 |
| industry | Organization | 100.27 | 1 | <0.001 |
| industry | Application | 36.07 | 1 | <0.001 |
| industry | Achievement | 26.88 | 1 | <0.001 |
| time within year z | Technology | 328.27 | 1 | <0.001 |
| time within year z | Achievement | 100.08 | 1 | <0.001 |
| time within year z | Environment | 87.35 | 1 | <0.001 |
| time within year z | Application | 77.43 | 1 | <0.001 |
| time within year z | Organization | 14.02 | 1 | <0.001 |
| time global z | Technology | 848.23 | 1 | <0.001 |
| time global z | Achievement | 392.88 | 1 | <0.001 |
| time global z | Environment | 139.97 | 1 | <0.001 |
| time global z | Application | 101.45 | 1 | <0.001 |
| time global z | Organization | 1.10 | 1 | 0.295 |

**Table S11. Ownership latent-mean difference under four specifications**

| Specification | Freed parameters | Difference | SE | Standardized | Ratio to nested model |
|---|---|---|---|---|---|
| scalar (full, rejected) | – | 0.150 | 0.021 | 0.238 | 1.233 |
| partial scalar, intercept only | g_env intercept | 0.119 | 0.021 | 0.188 | 0.982 |
| partial scalar given partial metric | g_tech loading + g_env intercept | 0.121 | 0.019 | 0.189 | 1.000 |
| raw unit-weighted composite | – | 0.146 | – | 0.236 | 1.203 |

Positive values indicate that non-state firms exceed state firms.

### S2.5 Measurement properties of the alternative keyword factor

**Table S12. Keyword factor, standardized loadings**

| Indicator | Std. loading | SE | Uniqueness |
|---|---|---|---|
| AITechnology | 0.677 | 0.040 | 0.542 |
| CloudComputingTech | 0.525 | 0.038 | 0.725 |
| BigDataTechnology | 0.763 | 0.036 | 0.417 |
| DigitalTechApplication | 0.367 | 0.038 | 0.865 |

**Table S13. Keyword factor, fit and reliability**

| Quantity | Value |
|---|---|
| CFI | 0.978 |
| TLI | 0.934 |
| RMSEA | 0.083 |
| SRMR | 0.025 |
| AVE | 0.363 |
| CR | 0.681 |
| omega | 0.675 |
| Mean inter-item r | 0.338 |

**Table S14. Cross-method agreement**

| Quantity | Value |
|---|---|
| HTMT | 0.771 |
| latent factor correlation | 0.781 |
| latent factor correlation se | 0.019 |
| factor score correlation pearson | 0.895 |
| factor score correlation spearman | 0.963 |
| unit weighted composite correlation pearson | 0.535 |
| unit weighted composite correlation spearman | 0.630 |
| nobs | 18,633 |
| n firms | 4,954 |
| year min | 2,021 |
| year max | 2,024 |

## §S2.6 Software and reproduction

R 4.5.2; lavaan 0.6.21; semTools 0.5.8. Estimation uses estimator="MLR" with cluster="stkcd". Reported chi-square values are the Yuan–Bentler $T_2^*$ statistic that lavaan returns under estimator="MLR" (see §S6.6) and standard errors are firm-clustered sandwich estimates. Fit indices are lavaan's robust variants computed from the scaled statistic (cfi.robust, tli.robust, rmsea.robust); SRMR is the standard unscaled value. Reliability omega is semTools::compRelSEM; AVE and CR are computed from the standardized solution; HTMT is semTools::htmt on standardized indicators.

Scripts, in execution order:

- run_reviewer_extensions.R — reproduction lock, panel structure, AVE/CR, two-factor CFA, score tests, partial-metric ladders, cross-method agreement, keyword-factor properties
- run_nested_partial.R — nested partial-metric to partial-scalar sequence
- run_latent_means.R — latent-mean comparison across specifications
- run_robustness.R — split-sample cross-validation and industry-switcher robustness
- make_figure2.R — Figure 2 and Supplementary Figure S1
- build_supplementary.R — this document

---

## §S3 Cross-validation of the partial-invariance specification search

Which loading to free was determined by score tests on the constrained model, not by prior theory. To bound the resulting concern we split the panel at the firm level (seed 20260818) and repeated the search independently in each half.

**Table S15. Split-sample cross-validation**

| Partition | Half | Rung | Freed | CFI | Delta CFI vs configural |
|---|---|---|---|---|---|
| ownership | A | configural | – | 0.9573 | 0.0000 |
| ownership | A | metric | – | 0.9491 | -0.0082 |
| ownership | A | partial metric | Technology | 0.9570 | -0.0003 |
| ownership | B | configural | – | 0.9507 | 0.0000 |
| ownership | B | metric | – | 0.9420 | -0.0087 |
| ownership | B | partial metric | Technology | 0.9502 | -0.0005 |
| industry | A | configural | – | 0.9615 | 0.0000 |
| industry | A | metric | – | 0.9243 | -0.0371 |
| industry | A | partial metric | Environment | 0.9530 | -0.0085 |
| industry | B | configural | – | 0.9484 | 0.0000 |
| industry | B | metric | – | 0.9144 | -0.0340 |
| industry | B | partial metric | Environment | 0.9415 | -0.0068 |
| time within year z | A | configural | – | 0.9800 | 0.0000 |
| time within year z | A | metric | – | 0.9664 | -0.0136 |
| time within year z | A | partial metric | Technology | 0.9720 | -0.0080 |
| time within year z | B | configural | – | 0.9727 | 0.0000 |
| time within year z | B | metric | – | 0.9545 | -0.0182 |
| time within year z | B | partial metric | Technology | 0.9632 | -0.0095 |

**Table S16. Agreement of the independent searches**

| Partition | Selected in half A | Selected in half B | Agreement |
|---|---|---|---|
| ownership | g_tech | g_tech | Yes |
| industry | g_env | g_env | Yes |
| time within year z | y_tech | y_tech | Yes |

## §S4 Robustness to industry-classification changes

295 of the 5,267 firms change industry classification during the panel. The industry conclusion is unchanged under three treatments of those firms.

**Table S17. Industry ladder under three classification treatments**

| Treatment | Rung | Firms | Firm-years | CFI | Delta CFI vs configural |
|---|---|---|---|---|---|
| as submitted time varying | configural | 5,267 | 46,799 | 0.9561 | 0.0000 |
| as submitted time varying | metric | 5,267 | 46,799 | 0.9200 | -0.0361 |
| as submitted time varying | partial metric free env | 5,267 | 46,799 | 0.9482 | -0.0079 |
| exclude switching firms | configural | 4,972 | 43,181 | 0.9542 | 0.0000 |
| exclude switching firms | metric | 4,972 | 43,181 | 0.9175 | -0.0367 |
| exclude switching firms | partial metric free env | 4,972 | 43,181 | 0.9451 | -0.0091 |
| first year fixed class | configural | 5,267 | 46,799 | 0.9557 | 0.0000 |
| first year fixed class | metric | 5,267 | 46,799 | 0.9314 | -0.0244 |
| first year fixed class | partial metric free env | 5,267 | 46,799 | 0.9482 | -0.0075 |

## §S5 Sector comparison under partial invariance (added in the second revision)

**Table S19. Between-sector variance share of each sub-dimension**

| Sub-dimension | Between-sector share, 19 CSRC sections | Between-sector share, manufacturing vs non-manufacturing | Mean difference (mfg − non-mfg, SD units) |
|---|---|---|---|
| Environment | 0.189 | 0.1375 | +0.785 |
| Technology | 0.164 | 0.0060 | -0.164 |
| Strategy | 0.128 | 0.0075 | -0.183 |
| Application | 0.116 | 0.0033 | -0.122 |
| Organization | 0.050 | 0.0001 | +0.015 |
| Achievement | 0.046 | 0.0004 | +0.044 |

*Note.* Shares are $\eta^2$ from one-way analyses of variance on the standardized indicators (N = 46,799 firm-years). Environment support accounts for 13.8% of its variance between the manufacturing and non-manufacturing groups; the next largest share is 0.8%. It is also the indicator whose loading and intercept the partial-invariance model frees.

**Table S20. Sector difference in latent means under four specifications**

| Specification | Freed parameters | Difference (mfg − non-mfg) | SE | Standardized |
|---|---|---|---|---|
| Raw unit-weighted composite | — | +0.063 | — | +0.102 |
| Full scalar (rejected) | — | -0.033 | 0.024 | -0.051 |
| Partial scalar, intercept only | g_env intercept | -0.130 | 0.022 | -0.198 |
| **Partial scalar given partial metric** | g_env loading + g_env intercept | -0.131 | 0.023 | -0.196 |

*Note.* The raw composite reports manufacturing firms as more digitally transformed; the partial-invariance model reports them as less. Manufacturing firms score lower on four of the six sub-dimensions, including the three that load most strongly, and higher only on environment support (+0.785 SD). A loading-weighted average omitting environment support gives −0.110, close to the model-based −0.131. Group order was read from lavInspect(fit,"group.label") rather than assumed.

---

## §S6 Analyses added in the third revision (Guest Associate Editor)

All results in this section were produced by repro_editor/E_*.R, which begins from the same reproduction lock as the earlier evidence layer (N = 46,799; CFI = 0.959267096; RMSEA = 0.063665008) and stops if it fails. Random splits use set.seed(20260905).

### S6.1 Missing data within the index coverage window

**Table S21. Missingness by year, ownership, and sector, and observed-versus-unobserved balance**

**Panel A — accounting**

| Quantity | Value |
|---|---|
| Source panel rows (2001–2024) | 59,241 |
| Dropped by listwise deletion on the six sub-dimensions | 12,442 |
| …of which fall in 2001–2010 (before the index begins) | 12,192 (98.0%) |
| …of which fall in 2011–2024 | 250 (2.0%) |
| Rows in the 2011–2024 window | 47,049 |
| Observed in the window | 46,799 (**99.5%**) |

**Panel B — within-window missing rates**

| Group | N | Missing rate |
|---|---|---|
| Non-state firms | 31,405 | 0.69% |
| State firms | 15,644 | 0.21% |
| Non-manufacturing | 15,767 | 0.36% |
| Manufacturing | 31,282 | 0.62% |
| 2011–2023 (each year) | — | 0.00%–0.10% |
| 2024 | 5,051 | 4.61% |

**Panel C — observed versus unobserved firm-years, 2011–2024**

| Variable | Mean, observed | Mean, unobserved | Standardized difference |
|---|---|---|---|
| Size (log assets) | 22.211 | 20.636 | +1.411 |
| Listing age (log) | 2.050 | 1.191 | +1.092 |
| Institutional ownership | 0.427 | 0.246 | +0.743 |
| Board size (log) | 2.109 | 2.016 | +0.469 |
| Leverage | 0.413 | 0.324 | +0.455 |
| Sales growth | 0.136 | 0.038 | +0.309 |
| Firm age (log) | 2.959 | 3.023 | −0.215 |
| Asset turnover | 0.624 | 0.575 | +0.127 |
| Cash flow | 0.046 | 0.051 | −0.078 |
| ROA | 0.037 | 0.040 | −0.046 |
| Largest shareholder | 0.337 | 0.343 | −0.043 |
| Board independence | 37.75 | 37.90 | −0.029 |
| Tobin's Q | 1.984 | 2.037 | −0.045 |
| ROE | 0.054 | 0.055 | −0.009 |

*Note.* The 12,442 rows removed by listwise deletion are almost entirely a coverage boundary, not attrition: 98.0% precede the first year the database scores annual reports on these dimensions. Within the window the index actually covers, coverage is 99.5%. The 250 unobserved firm-years are smaller and much more recently listed, consistent with new listings whose 2024 reports were not yet scored at extraction; they are 0.5% of the window.

**Table S22. Inverse-probability-weighted sensitivity analysis**

| Partition / model | CFI | ΔCFI (prev.) | ΔCFI (config.) | Latent contrast | SE |
|---|---|---|---|---|---|
| **Ownership** configural | 0.9582 | — | — | — | — |
| partial metric (free Technology loading) | 0.9570 | −0.0012 | −0.0012 | — | — |
| partial scalar \| partial metric | 0.9505 | −0.0066 | −0.0078 | −0.127 (state lower) | 0.019 |
| **Industry** configural | 0.9599 | — | — | — | — |
| partial metric (free Environment loading) | 0.9520 | −0.0079 | −0.0079 | — | — |
| partial scalar \| partial metric | 0.9464 | −0.0057 | −0.0136 | −0.128 (manufacturing lower) | 0.023 |

*Note.* Selection model: logit of index coverage on size, leverage, ROA, asset turnover, sales growth, board size, board independence, largest-shareholder share, institutional ownership, listing age, firm age, Tobin's Q, and cash flow, estimated within 2011–2024; weights are inverse fitted probabilities, normalized to mean one and trimmed at the 99th percentile (realized range 0.996–1.054). Because within-window coverage is 99.5%, the weights are near-uniform by construction and the analysis is a check that this is so rather than a correction of substance. These models retain `cluster="stkcd"` alongside sampling.weights, so the standard errors are cluster-robust; an earlier version of this table omitted the clustering, which left the point estimates and every decision unchanged but understated the standard errors by roughly a factor of two and a half. Every decision matches the unweighted analysis; latent contrasts differ by at most 0.006 SD.

### S6.2 Dimensionality beyond principal components

**Table S23. Parallel analysis on the six sub-dimensions**

| Position | Observed component eigenvalue | Simulated | Observed factor eigenvalue | Simulated |
|---|---|---|---|---|
| 1 | 2.372 | 1.015 | 1.749 | 0.191 |
| 2 | 1.002 | 1.008 | 0.115 | 0.010 |
| 3 | 0.829 | 1.003 | 0.079 | 0.004 |
| 4 | 0.735 | 0.998 | 0.010 | 0.000 |
| 5 | 0.552 | 0.992 | −0.068 | −0.005 |
| 6 | 0.510 | 0.985 | −0.136 | −0.011 |

*Note.* 100 simulated datasets, N = 46,799. One component is retained; four factors are nominally retained, but factors 2–4 have eigenvalues of 0.115, 0.079, and 0.010 against a first factor of 1.749 and are not substantively interpretable.

**Table S24. Exploratory solution in the discovery half and confirmation in the validation half**

**Panel A — two-factor exploratory solution, discovery half (maximum likelihood, oblimin)**

| Sub-dimension | ML1 | ML2 |
|---|---|---|
| Strategy | 0.656 | −0.025 |
| Technology | 0.665 | 0.049 |
| Organization | 0.002 | **0.997** |
| Environment | 0.412 | 0.015 |
| Achievement | 0.350 | −0.136 |
| Application | 0.654 | −0.004 |

Factor correlation 0.363. One-factor RMSEA 0.061, TLI 0.934; two-factor RMSEA 0.056, TLI 0.944; the three-factor solution is just identified (df = 0).

**Panel B — confirmation in the held-out half (N = 23,483 firm-years)**

| Model | CFI | TLI | RMSEA | SRMR | Factor correlation |
|---|---|---|---|---|---|
| One factor | 0.956 | 0.927 | 0.068 | 0.030 | — |
| Two factors, enabling versus realization | 0.956 | 0.918 | 0.072 | 0.030 | 0.966 |
| Two factors, principal-components axis (organization + achievement) | 0.973 | 0.949 | 0.057 | 0.022 | **3.765 (inadmissible)** |

*Note.* The exploratory second factor is defined by a single indicator and is therefore not a substantive dimension. The better-fitting principal-components structure returns a factor correlation above unity and is inadmissible. No admissible low-dimensional alternative improves on the single factor.

### S6.3 Panel structure

**Table S25. Indicator intraclass correlations**

| Sub-dimension | Between-firm variance | Within-firm variance | ICC |
|---|---|---|---|
| Environment | 0.918 | 0.082 | **0.918** |
| Strategy | 0.751 | 0.249 | 0.751 |
| Technology | 0.608 | 0.392 | 0.608 |
| Application | 0.503 | 0.498 | 0.503 |
| Organization | 0.249 | 0.751 | 0.249 |
| Achievement | 0.180 | 0.820 | 0.180 |

*Note.* One-way random-effects decomposition of the globally standardized indicators, firm as the grouping unit (5,267 firms,

46,799 firm-years). The pooled firm-year covariance structure is dominated by stable firm differences, most extremely for environment support, which is also the indicator whose non-invariance drives the sector result.

### S6.4 Where the sector contrast comes from

**Table S26. Indicator contributions to the sector contrast**

| Sub-dimension | Mean difference (mfg − non-mfg, SD) | Configural loading, non-mfg | Configural loading, mfg | Pooled loading | Contribution to unit-weighted composite | Contribution to loading-weighted score |
|---|---|---|---|---|---|---|
| Strategy | −0.183 | 0.645 | 0.662 | 0.643 | −0.030 | −0.038 |
| Technology | −0.164 | 0.783 | 0.658 | 0.710 | −0.027 | −0.038 |
| Organization | +0.015 | 0.476 | 0.303 | 0.365 | +0.003 | +0.002 |
| Environment | **+0.785** | 0.658 | 0.500 | 0.409 | **+0.131** | **+0.104** |
| Achievement | +0.044 | 0.351 | 0.263 | 0.297 | +0.007 | +0.004 |
| Application | −0.122 | 0.682 | 0.618 | 0.665 | −0.020 | −0.026 |
| **Total** | | | | 3.089 | **+0.063** | **+0.008** |

*Note.* Group order read from lavInspect(fit,"group.label"), which returned 0|1, so column 3 is non-manufacturing and column 4 manufacturing. Under equal weights, environment support contributes +0.131 of the composite's +0.063 total, so all other indicators combined contribute −0.068. Under loading weights its contribution falls to +0.104 of a total of +0.008.

**Table S27. Alternative scores and MIMIC specifications**

**Panel A — sector contrast under four scoring rules (composite SD units)**

| Score | mfg − non-mfg | t |
|---|---|---|
| Unit-weighted composite (the published rule) | **+0.102** | 10.06 |
| Loading-weighted score | +0.012 | 1.18 |
| Empirical factor score | −0.051 | −4.96 |
| Loading-weighted score excluding environment support | −0.160 | −15.40 |

**Panel B — MIMIC models (no group structure)**

| Model | Latent sector effect | SE | p | CFI | RMSEA | SRMR |
|---|---|---|---|---|---|---|
| Latent factor on sector only | −0.040 | 0.025 | 0.116 | 0.769 | 0.136 | 0.071 |
| …plus direct effect of sector on environment support | **−0.134** | 0.023 | <0.001 | 0.956 | 0.061 | 0.027 |
| …plus direct effect on technology | −0.124 | 0.024 | <0.001 | 0.956 | 0.064 | 0.027 |

Direct effect of sector on environment support: +0.877 (SE 0.019) in the second model, +0.870 (SE 0.020) in the third; the direct effect on technology is −0.031 (SE 0.019, $p = 0.105$).

*Note.* The MIMIC specification shares no structure with the partial multi-group model and yet returns essentially the same latent sector effect (−0.134 versus −0.131). Without a direct path from sector to environment support the model fits badly and the latent effect is indistinguishable from zero, which is the same finding read through a different lens: the sector difference in environment support is not construct variance.

### S6.5 Discovery and validation

**Table S28. Frozen specifications fitted in the untouched validation half**

Firms (not firm-years) were split at random into 2,633 discovery and 2,634 validation firms. Specifications were selected by score tests in the discovery half, frozen, and fitted in the validation half with no further search.

| Partition | Frozen loadings freed | Frozen intercepts freed | Sample | Configural CFI | Metric ΔCFI | Prespecified partial metric ΔCFI | Prespecified partial scalar ΔCFI (prev. / config.) | Prespecified partial scalar ΔRMSEA (prev.) | Latent contrast |
|---|---|---|---|---|---|---|---|---|---|
| Ownership | none | Environment | discovery | 0.9555 | −0.0071 | — | −0.0064 / −0.0136 | -0.0013 | +0.132 |
| Ownership | none | Environment | **validation** | 0.9499 | −0.0113 | — | **−0.0090 / −0.0203** | **-0.0008** | **+0.107** |
| Industry | Environment, Technology | Environment | discovery | 0.9561 | −0.0383 | −0.0088 | −0.0034 / −0.0122 | -0.0038 | −0.124 |
| Industry | Environment, Technology | Environment | **validation** | 0.9538 | −0.0333 | **−0.0040** | **−0.0072 / −0.0112** | **-0.0014** | **−0.138** |
| Time, within-year z | Technology, Achievement | none | discovery | 0.9785 | −0.0189 | −0.0096 | +0.0008 / −0.0088 | -0.0058 | +0.011 |
| Time, within-year z | Technology, Achievement | none | **validation** | 0.9735 | −0.0133 | −0.0069 | +0.0007 / −0.0062 | **-0.0062** | −0.011 |
| Time, global z | Technology, Achievement, Environment | Achievement, Organization | discovery | 0.9247 | −0.0429 | −0.0143 | −0.0002 / −0.0144 | -0.0059 | +0.141 |
| Time, global z | Technology, Achievement, Environment | Achievement, Organization | **validation** | 0.9226 | −0.0334 | −0.0109 | −0.0004 / −0.0113 | **-0.0062** | +0.126 |

*Note on score statistics.* Under se=”robust.cluster”, lavTestScore() warns that a cluster-robust score test is not implemented and falls back to the ordinary statistic. The univariate values reported throughout this supplement are therefore not cluster-robust. They are used only to *rank* candidate parameters for release, which is all the pre-specified freeing rule requires; no inference is drawn from them.

*Note.* Latent contrasts are signed so that the reported quantity is non-state minus state for ownership, manufacturing minus non-manufacturing for industry, and late minus early for time. (In lavaan’s own group indexing these are group(0) − group(1) for ownership and group(1) − group(0) for the other two, because state firms carry the code 1.) Signs were normalized using lavInspect(fit,”group.label”) for each fit separately, because lavaan orders groups by first appearance in the data and that order differs across halves. For ownership the metric constraint held in the discovery half, so no loading was frozen and the prespecified partial scalar model is nested under the fully constrained metric model. The discovery half selected environment support first for industry, the indicator the full-sample analysis also selects, and added technology because freeing environment alone did not clear the criterion in that half.

### Table S29. Two-wave longitudinal model, detail

Identification is by fixed factor: factor variances are set to unity and factor means to zero at each occasion in the configural model, and **all** loadings are freely estimated (std.lv=TRUE). An earlier version of this analysis fixed the factor variances while leaving lavaan's default marker-variable constraint in place, which double-scaled each occasion (df = 49 rather than 47) and produced a badly fitting configural model; that error is corrected here and every number below comes from the corrected specification.

**Panel A — full ladder**

| Model | $\chi^2$ | df | CFI | TLI | RMSEA | SRMR | ΔCFI (prev.) | ΔCFI (config.) |
|---|---|---|---|---|---|---|---|---|
| Configural | 480.8 | 47 | 0.9746 | 0.9644 | 0.0647 | 0.0408 | — | 0.0000 |
| Metric | 693.4 | 52 | 0.9614 | 0.9511 | 0.0759 | 0.0642 | -0.0132 | -0.0132 |
| **Partial metric** (free Technology loading) | 529.2 | 51 | 0.9712 | 0.9628 | 0.0662 | 0.0492 | -0.0034 | -0.0034 |
| Scalar (full, nested under metric) | 2877.2 | 57 | 0.8281 | 0.8010 | 0.1530 | 0.2072 | -0.1333 | -0.1465 |
| Scalar given partial metric | 2935.7 | 56 | 0.8280 | 0.7973 | 0.1544 | 0.2074 | -0.1432 | -0.1467 |
| Partial scalar, 1 intercept freed (Ach) | 1781.2 | 55 | 0.8946 | 0.8735 | 0.1220 | 0.1540 | -0.0766 | -0.0800 |
| Partial scalar, 2 freed (Ach, Tech) | 1331.4 | 54 | 0.9219 | 0.9046 | 0.1059 | 0.1563 | -0.0493 | -0.0527 |
| Partial scalar, 3 freed (Ach, Tech, Env) | 778.7 | 53 | 0.9553 | 0.9444 | 0.0809 | 0.0617 | -0.0159 | -0.0193 |
| **Partial scalar, 4 freed (Ach, Tech, Env, App)** | 539.3 | 52 | 0.9704 | 0.9624 | 0.0665 | 0.0512 | -0.0009 | -0.0043 |

**Panel B — score tests on the metric constraints (df = 1 each)**

| Constraint | $\chi^2$ |
|---|---|
| Technology loading | 246.1 |
| Achievement loading | 49.6 |
| Strategy loading | 46.6 |
| Environment loading | 31.0 |
| Organization loading | 24.2 |
| Application loading | 22.9 |

**Panel C — score tests on the intercept constraints, given partial metric (df = 1 each)**

| Constraint | $\chi^2$ |
|---|---|
| Achievement intercept | 1524.0 |
| Technology intercept | 1066.2 |
| Environment intercept | 800.1 |
| Application intercept | 548.2 |
| Strategy intercept | 4.9 |
| Organization intercept | 4.8 |

Four intercepts are strongly non-invariant; strategy and organization are not, and they are what remains as anchors once the other four are freed.

**Panel D — anchor dependence of the estimated change in level**

All fifteen two-anchor specifications, each freeing the other four intercepts on top of the partial metric model:

| Invariant anchors | CFI | ΔCFI vs partial metric | Satisfies criterion | Latent change (late − early) | SE |
|---|---|---|---|---|---|
| Environment + Achievement | 0.9425 | -0.0287 | no | **+2.411** | 0.174 |
| Organization + Achievement | 0.9620 | -0.0093 | **yes** | **+1.759** | 0.090 |
| Technology + Achievement | 0.8964 | -0.0749 | no | **+1.661** | 0.134 |
| Strategy + Achievement | 0.9486 | -0.0227 | no | **+1.532** | 0.076 |
| Achievement + Application | 0.9206 | -0.0506 | no | **+1.417** | 0.095 |
| Strategy + Organization | 0.9704 | -0.0009 | **yes** | **+0.904** | 0.028 |
| Strategy + Environment | 0.9396 | -0.0317 | no | **+0.901** | 0.034 |
| Strategy + Application | 0.9585 | -0.0127 | no | **+0.681** | 0.021 |
| Strategy + Technology | 0.9410 | -0.0302 | no | **+0.600** | 0.021 |
| Organization + Application | 0.9596 | -0.0116 | no | **+0.522** | 0.042 |
| Technology + Organization | 0.9525 | -0.0187 | no | **+0.301** | 0.024 |
| Technology + Application | 0.9704 | -0.0009 | **yes** | **+0.213** | 0.015 |
| Technology + Environment | 0.9586 | -0.0127 | no | **-0.017** | 0.019 |
| Environment + Application | 0.9570 | -0.0142 | no | **-0.055** | 0.019 |
| Organization + Environment | 0.9350 | -0.0362 | no | **-0.070** | 0.022 |

*Note.* The three specifications that satisfy the ΔCFI criterion imply latent changes of +0.213, +0.904, and +1.759 standard deviations. The full range across all fifteen is -0.070 to +2.411. For reference the raw unit-weighted composite rises by +0.278 indicator-standard-deviation units over the same firms and periods. **This table is the reason the paper reports no estimate of temporal change.** The partial scalar model is not rejected; it is underdetermined, and the fit criteria cannot choose among specifications that disagree eight-fold.

**Panel E — latent change under the models that do not satisfy the criterion, reported for completeness**

| Specification | Estimate | SE |
|---|---|---|
| Full scalar (ΔCFI = -0.1333 vs metric) | +0.930 | 0.053 |
| Scalar given partial metric (ΔCFI = -0.1432) | +0.934 | 0.066 |
| Partial scalar, four intercepts freed, anchors Strategy + Organization | +0.904 | 0.028 |

**Table S30. Joint two-factor model underlying the cross-method comparison**

| Quantity | Value |
|---|---|
| CFI (robust) | 0.876 |
| TLI (robust) | 0.836 |
| RMSEA (robust) | 0.082 [0.077, 0.087] |
| SRMR | 0.049 |
| Largest modification indices | Organization Achievement (1471.6); Application digital-technology-application keyword (580.4); AI big-data keyword (534.2); keyword factor =~ Technology (424.1) |

*Note.* The latent correlation of 0.781 used in Section 4.6 comes from this model. Its fit is moderate and its modification indices point to cross-family residual association, so the attenuation comparison in Section 4.6 is reported as descriptive and is not treated as excluding an aggregation-specific method effect.

**Table S31. Ownership, one observation per firm: full intercept search**

| Model | CFI | RMSEA | ΔCFI (prev.) | Non-state − state |
|---|---|---|---|---|
| Configural | 0.9119 | 0.1192 | — | — |
| Partial metric (free Technology loading) | 0.9047 | 0.1121 | −0.0072 | — |
| Partial scalar, no intercept freed | 0.8493 | 0.1272 | −0.0553 | +0.116 |
| Partial scalar, free Strategy | 0.8740 | 0.1186 | −0.0307 | +0.166 |
| Partial scalar, free Strategy + Organization | 0.8869 | 0.1146 | −0.0178 | +0.130 |
| **Partial scalar, free Strategy + Organization + Environment** | 0.9022 | 0.1087 | **−0.0025** | **+0.099** |

*Note.* The between-firm analysis reproduces the direction of the ownership contrast and selects the same non-invariant loading as the pooled analysis, but requires three freed intercepts rather than one, and its configural model fits poorly in absolute terms (RMSEA = 0.119). Section 4.3 states the conclusion this supports and the multilevel model it would take to settle the difference.

### S6.6 Estimator and test terminology as produced by the software

| Field | Value returned by lavaan 0.6.21 |
|---|---|
| estimator | ML |
| test | standard, **yuan.bentler.mplus** |
| se | **robust.cluster** |
| information | observed |
| Scaling correction factor, pooled one-factor model | 3.198 |
| R version | 4.5.2 (2025-10-31) |

*Note.* estimator=”MLR” in the model call produces maximum-likelihood estimates with the Yuan–Bentler $T_2^*$ test statistic in its Mplus variant and, with cluster=”stkcd”, cluster-robust standard errors. It does not produce a Satorra–Bentler scaled statistic. The manuscript now reports these labels rather than a generic robust-SEM description.

## S6.7 Contents of the reproduction deposit

The analysis code and aggregate supporting outputs are archived in Zenodo (version 1.0.0), DOI: 10.5281/zenodo.22897032. The archive contains baseline, original-submission, reviewer-revision and final editorial-revision scripts, their aggregate outputs, and proof-stage figure-generation materials. README.md and RESULT_MAP.md explain execution order and which later results supersede historical outputs; DATA_REQUIREMENTS.md documents input requirements and access restrictions; VALIDATION.md reports the checks performed during packaging.

The archive does not contain licensed CSMAR firm-level data or the estimation panel. Re-estimation requires independently authorized CSMAR access and reconstruction of the input panel; the upstream export/merge pipeline is not included. Aggregate outputs are supplied for inspection and figure generation and do not substitute for the microdata needed for robust, clustered or resampling analyses.

Code is licensed under MIT; documentation and aggregate outputs are licensed under CC BY 4.0. Neither license grants rights to third-party CSMAR data.

**Analysis script inventory**

| Round | Scripts |
|---|---|
| Original submission | `lavaan_tier0.R`, `lavaan_tier1.R`, `cfa_invariance.do` |
| First and second revisions (reviewer response) | `run_reviewer_extensions.R`, `run_robustness.R`, `run_nested_partial.R`, `run_latent_means.R`, `run_industry_latent_means.R`, `run_between_sector_variance.R`, `make_figure2.R`, `build_supplementary.R` |
| Third revision (this round) | `_common.R`, `E_A2_longitudinal_fixed.R`, `E_B_attenuation_contrib.R`, `E_B1b_attenuation_published.R`, `E_C_discovery_validation.R`, `E_D_icc_missing_dim.R`, `E_D2_missing_window.R`, `E_D3_ipw_efa_fix.R`, `E_D4_dim_validation.R`, `E_E_followups.R`, `make_figure2_v4.R` |

*Note.* `_common.R` carries the reproduction lock (N = 46,799; CFI = 0.959267096; RMSEA = 0.063665008) and is sourced by every script of the third round; each of those scripts aborts if the lock fails. Random splits use `set.seed(20260905)`.
